# PRMT5 inhibition impairs Fanconi Anemia pathway-mediated homologous recombination and enhances the antitumor efficacy of Temozolomide in glioblastoma

**DOI:** 10.1038/s41419-026-08739-5

**Published:** 2026-04-15

**Authors:** Shumpei Onishi, Sridharan Jayamohan, Ashis Chowdhury, Sarah Rivas, Yoshihiro Otani, Celine Ertekin, Jean-Paul Bryant, Sara A. Murphy, Kimberly A. Rivera-Caraballo, Stuart Walbridge, Bayu Sisay, Dragan Maric, Abdel Elkahloun, Kory Johnson, Desmond A. Brown, John D. Heiss, Ashish H. Shah, Tae Jin Lee, Sangamesh G. Kumbar, Ji Young Yoo, Andrew J. Brenner, Balveen Kaur, Gangadhara R. Sareddy, Yeshavanth Kumar Banasavadi-Siddegowda

**Affiliations:** 1 Surgical Neurology Branch, National Institute of Neurological Disorders and Stroke, National Institutes of Health, Bethesda, MD USA; 2 Department of Obstetrics and Gynecology, University of Texas Health San Antonio, San Antonio, TX USA; 3 Department of Neurosurgery, University of Texas Health Science Center at Houston, Houston, TX USA; 4 Georgia Cancer Center, Augusta University Medical Center, Augusta, GA USA; 5 Cancer Genetics Branch, National Human Genome Research Institute, National Institutes of Health, Bethesda, MD USA; 6 Flow and Imaging Cytometry Core Facility, NINDS, NIH, Bethesda, MD USA; 7 Bioinformatics Core, Information Technology Program, NINDS, NIH, Bethesda, MD USA; 8 Department of Growth and Development, College of Dentistry, University of Nebraska Medical Center, Omaha, NE USA; 9 Mays Cancer Center, University of Texas Health San Antonio, San Antonio, TX USA

**Keywords:** Cancer therapeutic resistance, CNS cancer

## Abstract

Despite multimodal therapy of surgical resection, radiation, and chemotherapy, glioblastoma patients show a dismal prognosis. Protein Arginine Methyltransferase 5 (PRMT5) is overexpressed in glioblastoma, and its inhibition imparts an anti-tumor effect. Tumor cells invariably develop resistance to Temozolomide (TMZ), the standard chemotherapeutic agent for glioblastoma. However, the mechanistic role of PRMT5 in treatment-resistant glioblastoma is unknown. Patient-derived glioma stem-like cells (GSCs), treated with PRMT5 inhibitor (LLY-283) or transfected with PRMT5-target-specific siRNA, were treated with TMZ and subjected to in vitro functional and mechanistic studies. The intracranial mouse xenograft model was used to test the in vivo antitumor efficacy of combination treatment. We found that PRMT5 inhibition increased the cytotoxic effect of TMZ in GSCs. Unbiased transcriptomic profiling revealed negative enrichment of DNA damage repair pathways, with prominent suppression of the Fanconi anemia (FA) pathway. PRMT5 inhibition abrogated the TMZ-induced G2/M cell cycle arrest. Importantly, combination treatment increased the DNA double-strand breaks (γH2AX foci) and enhanced the DNA damage (comet assay). Specifically, the LLY-283 treatment blocked the FA pathway-mediated homologous recombination repair in GSCs. In vivo, the LLY-283 and TMZ combination significantly curbs tumor growth and prolongs the survival of tumor-bearing mice. Furthermore, compared to monotherapy, there was a significant reduction in the proliferation marker Ki-67, while the apoptosis marker cleaved caspase 3 and the DNA damage response marker γH2AX were upregulated. Collectively, these findings identify PRMT5 as a critical regulator of the FA pathway in glioblastoma and demonstrate that PRMT5 inhibition potentiates TMZ efficacy by disrupting FA-dependent homologous recombination repair, indicating that the combination of PRMT5 inhibition and TMZ could be a novel therapeutic strategy for glioblastoma.

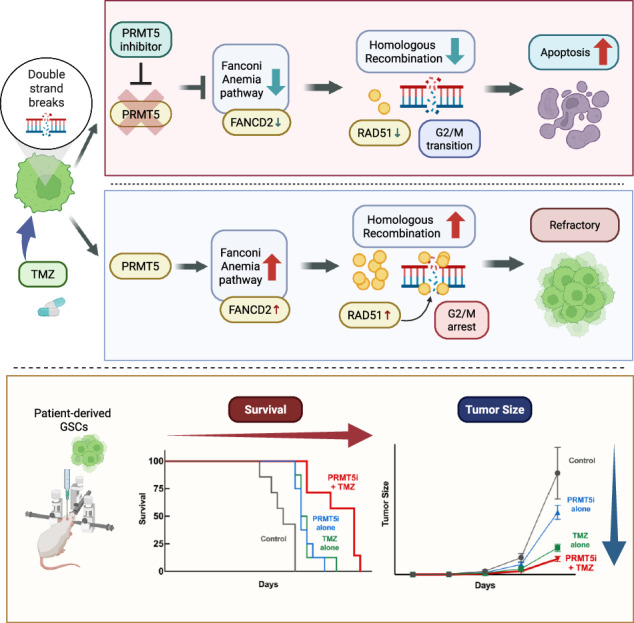

## Introduction

Glioblastoma is the most common malignant primary brain tumor, treated with a combination of maximum safe surgical resection followed by radiation and chemotherapy. Despite these efforts, median survival is around 15–20 months due to tumor progression and recurrence [1, 2]. Intra-tumoral heterogeneity and acquired therapy resistance have been implicated in treatment failure [3–5]. A key factor contributing to therapy resistance is the intrinsic DNA repair capacity of tumor cells, particularly glioma stem-like cells (GSCs) [6, 7] that exhibit dysregulated DNA damage response pathways [8–10].

Protein arginine methyltransferase 5 (PRMT5) regulates numerous cellular functions through the symmetric di-methylation of histone and non-histone arginine proteins. In glioblastoma, its expression is dysregulated [11, 12], and its inhibition imparts an anti-tumor effect [13]. PRMT5 is a druggable target, and several PRMT5 inhibitors, including LLY-283 are under investigation for glioblastoma treatment [14–16]. Additionally, PRMT5 inhibitors are currently in clinical trials (ClinicalTrials.gov; MRTX1719 [NCT06883747], TNG456 [NCT06810544], TNG908 [NCT05275478], PRT811 [NCT04089449], AMG193 [NCT05094336]). Recent studies indicate that PRMT5 regulates homologous recombination (HR) repair through methylation of RUVBL1 and histone arginine residues [17, 18]. It also controls DNA repair by regulating the histone-modifying enzymes through alternative splicing [19], activating epigenetic activators [20], and promoting non-homologous end joining (NHEJ) by stabilizing and methylating 53BP1 [21].

Temozolomide (TMZ), through alkylation of guanine at the O6 position, induces DNA damage [22]. From the time the anti-tumor efficacy of TMZ for newly diagnosed glioblastoma was validated, it has been the first-line chemotherapeutic intervention for glioblastoma [1]. However, TMZ resistance and subsequent recurrence/progression remain a significant challenge [23]. Primarily, TMZ resistance has been linked to the MGMT (O⁶-methylguanine-DNA methyltransferase) status of the tumor cells. But, recent studies have identified alternative factors that significantly contribute to TMZ-resistance: (i) Intrinsic ability of GSCs to repair TMZ-induced DNA damage [6, 24, 25], (ii) epigenetic modifications, and (iii) signaling cascade dysregulation [26] are some of the major contributors towards TMZ-resistance mechanisms, thus increasing the complexity of TMZ-resistance.

Given the enhanced intrinsic DNA repair ability of GSCs and the role of DNA repair mechanisms in TMZ resistance, we investigated whether PRMT5 inhibition could alter the TMZ resistance in GSCs by impairing DNA damage repair. Our study demonstrates that PRMT5 inhibition suppresses HR repair of glioblastoma, increases TMZ-induced DNA damage, and enhances the antitumor efficacy in both in vitro and in vivo glioblastoma tumor models.

## Results

### PRMT5 inhibition increases the sensitivity of TMZ in GSCs

**Fig. 1:**
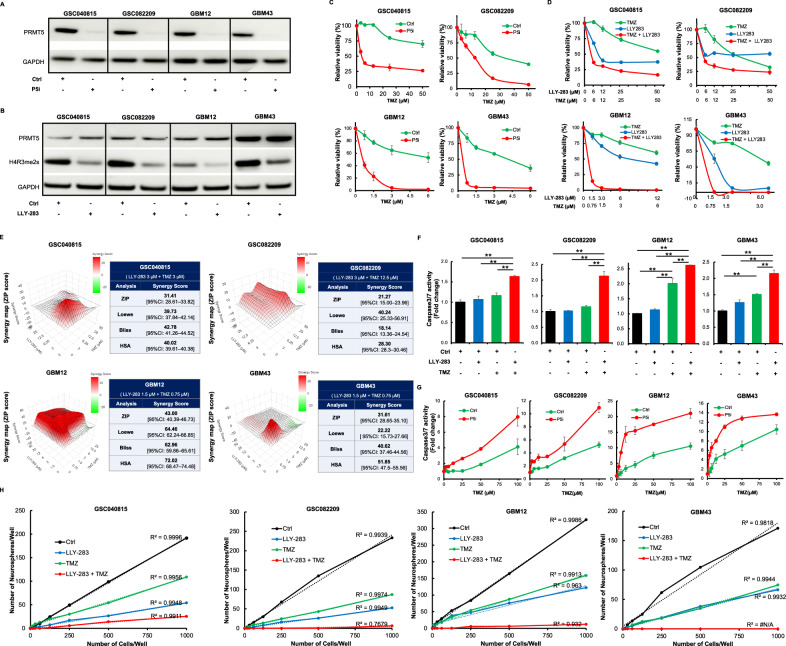
PRMT5 inhibition increases the sensitivity of TMZ in GSCs. **A** GSCs were transfected with scrambled (Ctrl) or PRMT5-target specific siRNA (P5i). 72 hours post-transfection, cells were probed for PRMT5 by western blot. **B** Indicated GSCs were treated with LLY-283 (50 µM). 3 days post-treatment, cells were collected and probed for PRMT5 marker H4R3 by western blot. **C** GSCs transfected with Ctrl or P5i were treated with increasing doses of TMZ. 4 days post-treatment, cells were subject to viability assay. **D** GSCs were treated with either TMZ and/or LLY-283. 4 days post-treatment, viability of the cells was measured by Cell-Titer Glo assay. **E** Based on the Fig. 1D data, the synergy score was calculated. Representative synergy score graph for ZIP model and the table for synergy scores across 4 analysis models are shown. **F** GSCs were treated with TMZ and/or LLY-283 for 48 hours, and caspase3/7 activity was measured. **G** PRMT5-intact and depleted GSCs were treated with increasing doses of TMZ, and caspase3/7 activity was measured 48 h post-treatment with TMZ. **H** Increasing number of indicated GSCs ranging from 15 to 1000 cells per well were seeded and treated with Ctrl (DMSO), LLY-283 (GSC040815 & GSC082209 with 50 µM and GBM12 & GBM43 with 3 µM), TMZ (GSC040815 & GSC082209 with 50 µM and GBM12 & GBM43 with 6 µM) or the combination of LLY-283 and TMZ. Seven days post-treatment, the number of neurospheres formed in each well was counted and plotted against the number of cells seeded. The thin dash line represents the linear trend. To compare the frequency of neurosphere formation, linear regression was performed as shown. *n* = 3 for all the experiments (** *p* ≤ 0.001).

To test if PRMT5 alters the effect of TMZ in glioblastoma, we used GSCs that are relatively TMZ-resistant (GSC040815 and GSC082209) and TMZ-sensitive (GBM12 and GBM43). While knockdown of PRMT5 was confirmed by probing for PRMT5 expression, inhibition of PRMT5 activity by LLY-283 was confirmed by the reduction in symmetrically di-methylated H4R3 (Figs. 1A, B & Suppl. Fig. 2). We treated PRMT5-depleted GSCs with increasing doses of TMZ (Fig. 1C). PRMT5 depletion reduced the EC50 value of TMZ by at least 25-fold. Furthermore, LLY-283 treatment reduced the EC50 value of TMZ from over 30 µM to under 5 µM in TMZ-resistant GSCs, and from 6 µM to under 1 µM in TMZ-sensitive GSCs (Fig. 1D). To further test if the combinatorial effect of TMZ and LLY-283 is synergistic, we analyzed Fig. 1D data for synergy score using Synergy Finder software (Fig. 1E). Synergy score was more than 20 in all four types of analysis and across all cell types tested. Thus, confirming that the combination of TMZ and LLY-283 has synergistic effect on the GSCs. To determine if the enhanced synergistic cytotoxic effect was due to apoptosis, we conducted caspase 3/7 activity assay (Fig. 1F, G). Combination therapy significantly increased caspase 3/7 activity. To test the effect of combination treatment on the self-renewal capacity of GSCs, we conducted neurosphere formation assay (Fig. 1H). As expected, PRMT5 inhibition with LLY-283 or TMZ treatment reduced the number of neurospheres. Most importantly, the combination therapy resulted in a significant reduction in neurosphere numbers compared to LLY-283 or TMZ treatment alone, indicating that the combination treatment suppresses the self-renewal of GSCs. These results confirm that PRMT5 inhibition sensitizes GSCs to TMZ and synergistically enhances TMZ-induced cytotoxicity.

### PRMT5 regulates DNA damage repair in GSCs

**Fig. 2:**
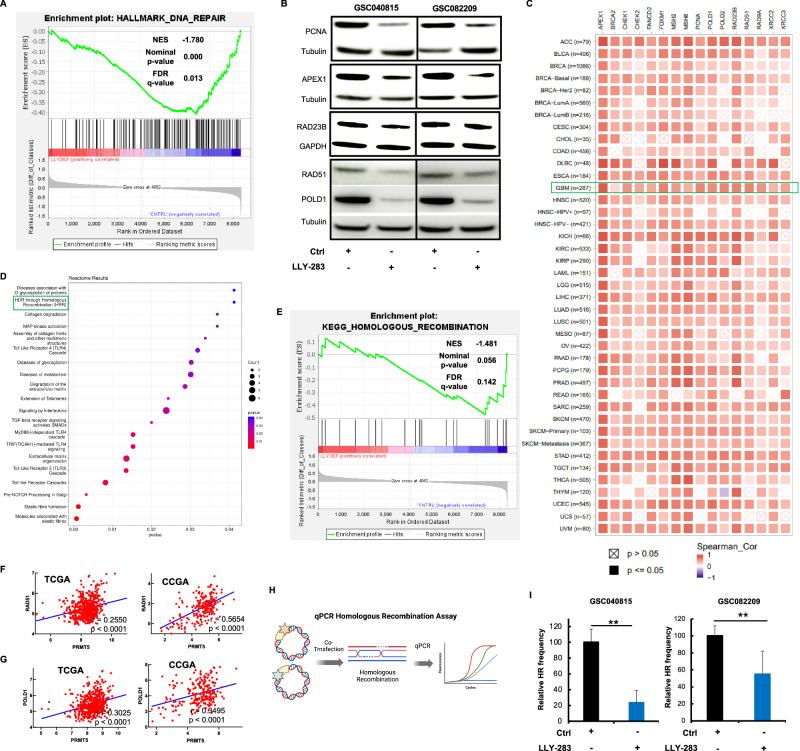
PRMT5 inhibition downregulated DNA damage repair genes in GSCs. **A** GSC082209 treated with Control (Ctrl/DMSO) or LLY-283 (50 µM) for 24 h were subjected to RNA sequencing. GSEA enrichment plot shows the negative enrichment of DNA damage repair genes in LLY-283 treated GSCs. **B** GSC040815 and GSC082209 treated with LLY-283 (50 µM) for 72 h were probed for indicated DNA damage repair proteins by western blot. **C** Heatmap generated using TIMER2.0 with data from The Cancer Genome Atlas (TCGA) showing the correlation between PRMT5 and DNA repair genes across multiple tumor types. **D** Top differentially expressed genes based on the RNA sequencing analysis. **E** GSEA enrichment analysis showing a negative correlation between LLY-283 treatment and HR repair gene set. **F**, **G** Scatter plot from Gliovis database showing correlation between PRMT5, and HR genes (RAD51 and POLD1) based on glioblastoma TCGA and CCGA data sets. p-value computed for each data set. **H** Schematic representation of qPCR-based HR assay protocol. **I** GSC040815 and GSC082209 treated with LLY-283 were subjected to HR activity. *n* = 3 for all the experiments. (** *p* ≤ 0.001).

To gain mechanistic insights into the combination effect, we performed RNA-sequencing analysis on the GSC082209 treated with LLY-283 or TMZ, or the combination. GSEA analysis revealed that LLY-283 treatment negatively enriched genes involved in DNA repair pathways (Fig. 2A and Suppl. Fig. 3A). Western blot analysis confirmed this finding, showing that LLY-283 treatment reduced the expression of DNA repair genes such as PCNA, RAD51, POLD1, APEX1 and RAD23B (Fig. 2B & Suppl. Fig. 4). To further explore the association between PRMT5 and DNA damage repair genes across various cancers, we analyzed the correlation between PRMT5 and DNA damage repair genes using TIMER2.0 with data from The Cancer Genome Atlas (TCGA). PRMT5 expression positively correlated with DNA damage repair genes across multiple tumor types, including glioblastoma (Fig. 2C). Additionally, the literature supports the link between PRMT5 and the DNA damage repair mechanism [17–21].

One of the top downregulated pathways with LLY-283 treatment is Homology-directed Repair (HDR) through HR (Fig. 2D). GSEA analysis showed negative enrichment for the HR genes (Fig. 2E and Suppl. Figure 3B). Analyzing the TCGA and CCGA patient databases, we found a positive correlation between PRMT5 expression and HR genes (RAD51 and POLD1) (Fig. 2F, G). To further confirm HR repair in the context of LLY-283 treatment in GSCs, we conducted the HR repair assay (Fig. 2H, I), and it showed a significant reduction in the HR repair in GSCs treated with LLY-283. These results together suggest that LLY-283 treatment negatively affects the DNA repair gene sets in general and HR in particular.

### PRMT5 inhibition enhances the TMZ-induced DNA damage

**Fig. 3:**
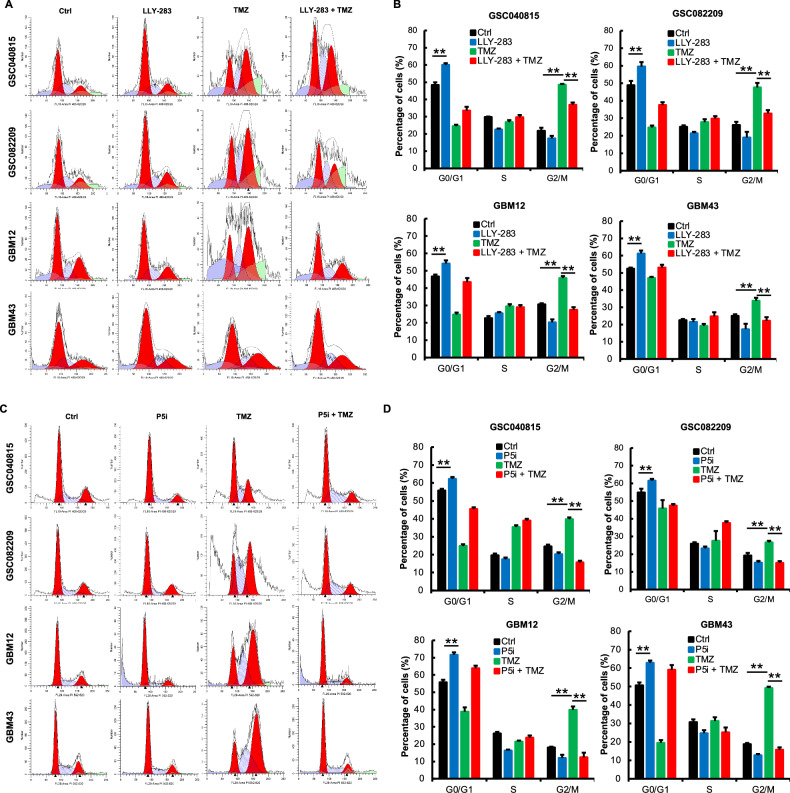

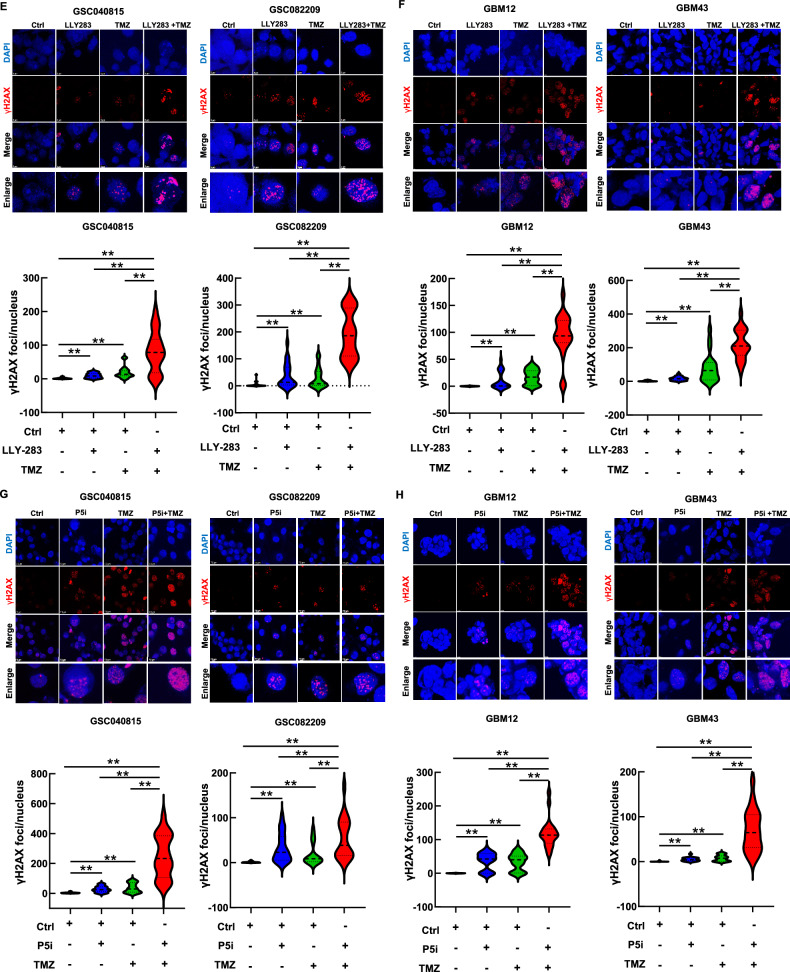

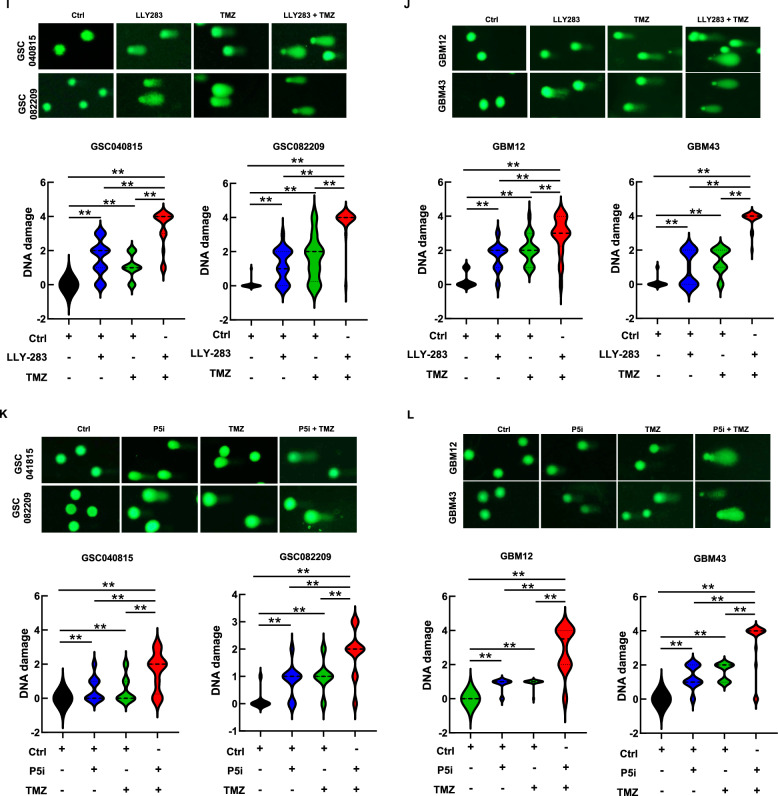
PRMT5 inhibition enhances the TMZ-induced DNA damage. **A**, **B** GSCs treated with LLY-283 (50 µM), TMZ (50 µM) or the combination of both for 48 hours were analyzed for the cell cycle progression using PI. **A** Representative cell cycle histogram for each treatment condition across all the cell types that were analyzed for cell cycle progression. **B** The graph represents the percentage of the cell population in each stage of the cell cycle (**p ≤ 0.001). **C**, **D** PRMT5-intact and depleted GSCs treated with TMZ (50 µM) for 48 h were analyzed for cell cycle progression using PI. **C** Representative cell cycle histogram for each treatment condition across all the cell types that were analyzed for cell cycle progression. **D** The graph represents the percentage of the cell population in each stage of the cell cycle (**p ≤ 0.001). **E**, **F** GSCs treated with LLY-283 and/or TMZ for 48 h as detailed in the methods section were probed for γH2AX foci by immunofluorescence and quantified. Upper panel: Representative microscopic images for γH2AX & DAPI. The last row of the images shows the representative enlarged image. Lower panel: Graph showing the quantification of the γH2AX foci (**p ≤ 0.001). **G**, **H** PRMT5 siRNA-transfected cells (P5i) treated with TMZ as detailed in the methods section were probed for γH2AX foci by immunofluorescence, and the number of foci was quantified. Upper panel: Representative microscopic images for γH2AX & DAPI. The last row of the images shows the representative enlarged merged image. Lower panel: Graph showing the quantification of the γH2AX foci. (**p ≤ 0.001). **I**, **J** GSCs treated with LLY-283 and/or TMZ for 48 h as detailed in the methods section were subjected to comet assay. Upper panel: Images of the representative comets across each treatment condition. Lower panel: DNA damage was graded/quantified from 0 to 4 based on the tail length and comet head size. (0 = no, 1= mild, 2 = moderate, 3 = high, 4= very high DNA damage. (**p ≤ 0.001). **K**, **L** GSCs transfected with P5i were treated with TMZ for 48 h and were subjected to comet assay. Upper panel: Images of the representative comets across each treatment condition. Lower panel: DNA damage was graded/quantified from 0 to 4 based on the tail length and comet head size. (0 = no, 1= mild, 2 = moderate, 3 = high, 4= very high DNA damage. n = 3 for all the experiments. (**p ≤ 0.001).

As the database analysis and RNA-seq results implicated the role of PRMT5 in DNA damage repair, we sought to explore the combination treatment in the context of DNA damage, response, and repair. TMZ induces DNA damage, causing G2/M cell cycle arrest as cells attempt to repair the damage [27, 28]. GSCs, with high DNA repair capacity, contribute to TMZ resistance [6, 7]. Hence, we tested whether PRMT5 inhibition affects the TMZ-induced G2/M arrest. PRMT5-inhibited GSCs were treated with TMZ, and cell cycle analysis was conducted 48 hours post-treatment (Fig. 3A–D). As expected, TMZ treatment induced the G2/M arrest. Interestingly, PRMT5 inhibition abrogated the G2/M arrest induced by TMZ, suggesting that PRMT5 may assist in repairing TMZ-induced DNA damage.

Further, we probed for γH2AX, a classical marker of DNA double-strand breaks (Fig. 3E, F). Sublethal doses of TMZ and LLY-283 increased the number of γH2AX foci compared to control. With the combination treatment, there was a significant increase in the number of foci, suggesting enhanced DNA damage. We also treated PRMT5-depleted GSCs with TMZ and probed for γH2AX foci. PRMT5 knockdown in combination with TMZ enhanced the γH2AX foci formation (Fig. 3G, H).

To further confirm that PRMT5 inhibitor-mediated downregulation of DNA repair enhances TMZ-mediated DNA damage, we conducted the comet assay. GSCs treated with LLY-283 and TMZ were subjected to the comet assay (Fig. 3I, J, Suppl. Figure 5I & J). Semi-quantitative analysis of DNA damage in the form of tail length and size of the comet head showed that with LLY-283 or TMZ treatment alone, there was a significant increase in DNA damage. With the combination of LLY-283 and TMZ, the DNA damage was robust. Also, there was enhanced DNA damage with the treatment of PRMT5-depleted GSCs with TMZ across all GSCs tested (Fig. 3K, L, Suppl. Figure 5K & 3L). Together, these results confirm that PRMT5 inhibition potentiates TMZ-induced DNA damage.

### LLY-283 blocks the TMZ-induced HR repair in GSCs

**Fig. 4:**
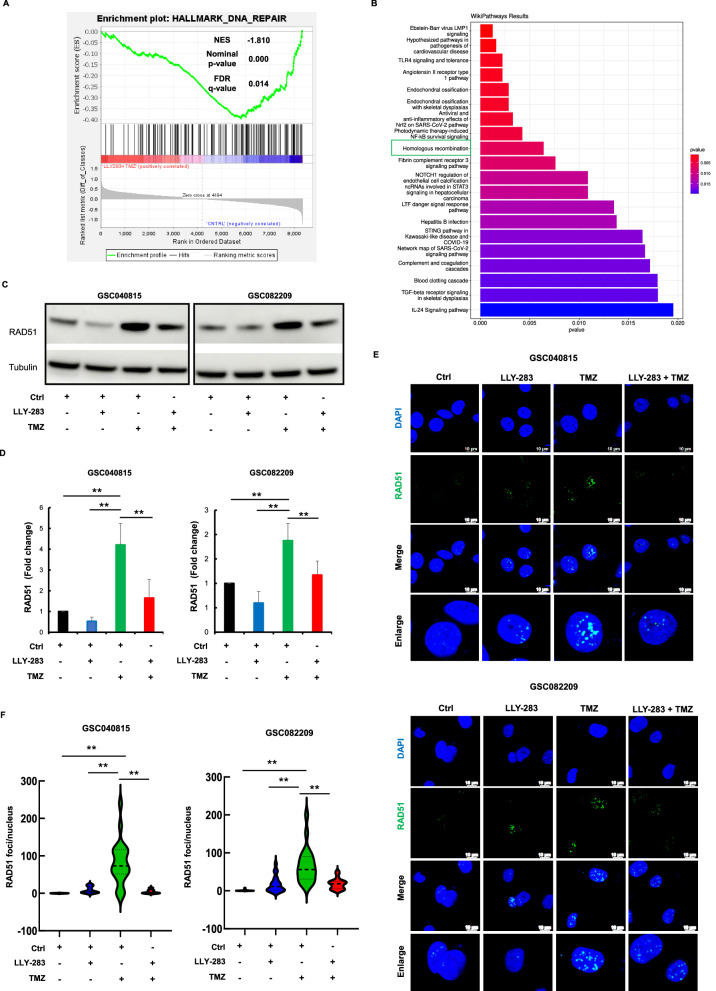
LLY-283 blocks the TMZ-induced HR repair in GSCs. **A** GSEA enrichment plot showing negative enrichment of DNA repair gene sets in GSC082209 treated with LLY-283 and TMZ combination. **B** Top gene ontology terms of differentially expressed genes pointing out HR. **C** GSC040815 and GSC082209 treated with LLY-283 (50 µM), TMZ (50 µM), or the combination of LLY-283 and TMZ, were probed for HR marker RAD51 by western blot. **D** Graph showing the quantification of RAD51 blots of panel **C** that were normalized to GAPDH (**p ≤ 0.001). **E**, **F** GSCs treated with LLY-283 and/or TMZ for 48 h were probed for RAD51 foci by immunofluorescence and quantified. **E** Representative microscopic images for RAD51 & DAPI. The last row of the images shows the representative enlarged images. **F** Graph showing the quantification of the RAD51 foci. *n* = 3 for all the experiments. (**p ≤ 0.001).

Having confirmed enhanced DNA damage with the combination therapy, we conducted gene enrichment analysis for the combination treatment condition (LLY-283 + TMZ) (Fig. 4A & Suppl. Figure 6). The combination treatment showed negative enrichments of DNA damage repair genes. Further, WikiPathways analysis (Fig. 4B) showed HR as one of the topmost pathways that were downregulated with the combination of LLY-283 and TMZ. To confirm this result, we probed for the HR marker RAD51 by western blot analysis (Fig. 4C, D & Suppl. Fig. 7). While TMZ increased the level of RAD51 protein, LLY-283 downregulated it. To further test if TMZ-induced enhanced RAD51 protein is associated with the functional activity of DNA repair, we measured RAD51 foci formation (Fig. 4E, F). TMZ treatment increased the number of foci/nuclei, LLY-283 treatment reduced the TMZ-induced RAD51 foci significantly, suggesting suppression of RAD51 activity by PRMT5 inhibition. These results confirm that LLY-283 blocks the TMZ-induced HR repair mechanism, thus enhancing the TMZ-induced DNA damage and subsequent sensitization of GSCs to TMZ.

### PRMT5 regulates Fanconi anemia pathway-induced TMZ resistance

**Fig. 5:**
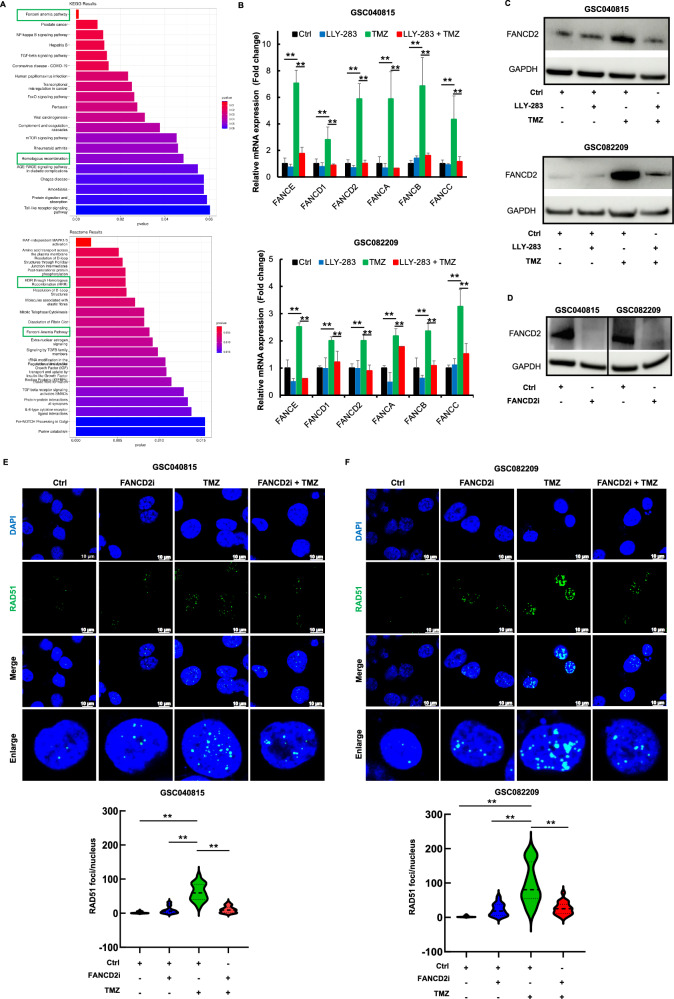
FA pathway regulates TMZ-induced HR repair in GSCs. **A** Top gene ontology terms of differentially expressed genes pointing out FA pathway and HDR through HR. **B** GSC040815 and GSC082209 treated with LLY-283 (50 µM), TMZ (50 µM), or the combination of LLY-283 and TMZ, were probed for FA genes by qPCR. **C** GSC040815 and GSC082209 treated with LLY-283 (50 µM), TMZ (50 µM), or the combination of LLY-283 and TMZ, were probed for FANCD2 by western blot. **D** GSC040815 and GSC082209 were transfected with FANCD2i. 72 hours post-transfection, FANCD2 knockdown was confirmed by western blot. **E, F** FANCD2-depleted GSCs were treated with TMZ (50 µM) or the combination. 48 h post-treatment, cells were probed for RAD51 foci by immunofluorescence. Upper panel: Representative microscopic images for RAD51 & DAPI. The last row of the images shows the representative enlarged images. Lower panel: Graph showing the quantification of the RAD51 foci. n = 3 for all the experiments except. (**p ≤ 0.001).

KEGG and Reactome enrichment analysis (Fig. 5A) showed Fanconi anemia (FA) pathway and HDR through HR were significantly downregulated with the combination of LLY-283 and TMZ. Prior studies have highlighted the crucial role of the FA pathway in conferring resistance towards DNA alkylating agents, including TMZ [29], and inhibition of FA pathway can sensitize tumor cells to radiation and chemotherapy [29]. Additionally, PRMT5 has been reported to regulate FA genes in cancer cells [30]. Based on this premise, we tested if PRMT5 regulates HR through FA pathway in the context of LLY-283/TMZ treatment.

Initially, we probed for the expression of FA genes by qPCR in the GSCs treated with LLY-283, TMZ, or the combination (Fig. 5B). TMZ treatment increased mRNA expression of all the FA genes tested by at least 2-fold and was reduced significantly with LLY-283 treatment, suggesting potential regulation of the FA pathway by PRMT5. Further, we probed FANCD2 expression by western blot, the key effector target of the FA pathway (Fig. 5C & Suppl. Fig. 8). While TMZ induced the expression of FANCD2, LLY-283 significantly reduced the TMZ-induced FANCD2 expression. Next, we transfected the GSCs with FANCD2-target-specific siRNA (FANCD2i) (Fig. 5D & Suppl. Fig. 8), treated them with TMZ, and probed for RAD51 foci formation (Fig. 5E, F). FANCD2i effectively downregulated TMZ-induced RAD51 foci formation, indicating that FA pathway inhibition blocks TMZ-induced HR repair, thereby enhancing DNA damage and sensitizing GSCs to TMZ. These findings suggest that PRMT5, through the FA pathway, reduces HR repair in TMZ-treated GSCs.

### In vivo, PRMT5 inhibition enhances the antitumor efficacy of TMZ

**Fig. 6:**
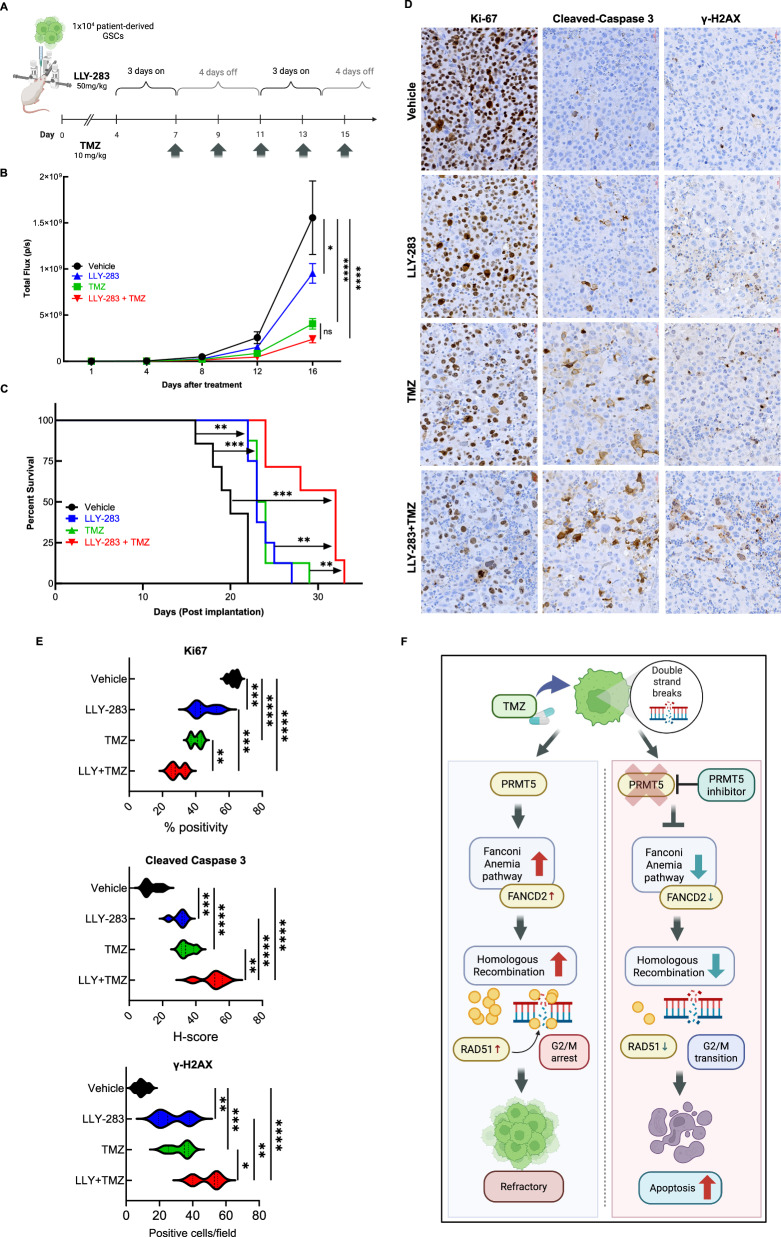
Combination of LLY-283 and TMZ enhances the in vivo antitumor efficacy. **A** Schematic representation of the in vivo study. **B** Mice were implanted with GSC040815 that expresses GFP-Luciferase and quantification of the tumour volume based on the luciferase images was generated during the study. **C** Post-implantation, mice were treated with different treatment conditions and the Kaplan–Meier survival curve was plotted at the end of the study. **D**, **E** Tumor tissues harvested at the end of the study were stained for Ki-67, γH2AX, and Cleaved Caspase3 by IHC. *p < 0.05, **p < 0.01, ***p < 0.001, ****p < 0.0001 by one-way ANOVA. **F** Schematic representation of the working model for the PRMT5-inhibition-triggered apoptosis in the TMZ-treated GSCs. *n* = 8. 8 mice were used in each treatment group.

To assess the effect of combination therapy on tumor growth and survival, we used an intracranial glioblastoma mouse model. GSC040815-Luc were implanted in the mice and were treated with LLY-283 and/or TMZ as detailed in Fig. 6A, materials and methods, and supplementary data. We followed tumor growth throughout the study (Fig. 6B). Treatment with LLY-283 and TMZ combination reduced the tumor growth significantly. Monotherapy with LLY-283 or TMZ increased the median survival of tumor-bearing mice from 20 days to 23 days (Fig. 6C). However, the median survival of mice in the combination treatment increased to 33 days, suggesting that the combination treatment has a better anti-tumor effect compared to LLY-283 or TMZ treatment alone. Further, analysis of tumor sections by IHC showed a significant reduction of proliferation marker Ki-67, substantial induction of apoptosis marker cleaved caspase3, and DNA double-strand break (DSB) marker γH2AX (Fig. 6D, E). These results together suggest that inhibition of PRMT5 significantly enhances the TMZ efficacy and improves the overall survival of glioblastoma tumor-bearing mice. The schematic representation (Fig. 6F) depicts the potential mechanism through which PRMT5 inhibition sensitizes GSCs for TMZ through the FA pathway.

## Discussion

The therapeutic outcome for glioblastoma is grave even with multimodal standard therapy that includes surgical resection followed by radiation and concurrent chemotherapy. Apart from tumor heterogeneity and activation of tumor escape pathways, the intrinsic ability of glioblastoma tumor cells to repair the damaged DNA induced by treatment plays a significant role in imparting radio- and/or chemotherapy resistance. The key to overcoming this issue is to explore the potential resistance mechanism and develop a therapeutic regimen that combines drugs that synergize with each other to produce additional anti-tumor efficacy at lower, less toxic doses.

PRMT5 acts as a critical regulator of DNA damage repair through multiple molecular pathways to stabilize the genomic DNA and facilitate the DNA repair processes. PRMT5 through methylation of RUVBL1 plays an important role in coordinating double-strand break by HR [17]. Further, evidence shows that PRMT5 mediates HR repair through histone arginine-methylation to maintain genomic stability [18]. PRMT5, in coordination with pICln acts as an epigenetic activator of DNA double-strand break repair genes [20]. Through the regulation of alternative splicing of histone-modifying enzymes, PRMT5 controls DNA repair [19]. With the infliction of DNA damage, PRMT5 promotes NHEJ DNA repair through methylation and stabilization of 53BP1 and is regulated by Src-mediated phosphorylation [21]. In this study, in the context of glioblastoma, our results show a negative correlation between PRMT5 inhibition and DNA repair pathways, thus reconfirming the pivotal role played by PRMT5 in DNA damage repair machinery.

TMZ, despite being the primary chemotherapeutic agent in the treatment of glioblastoma, the development of resistance to it remains a significant obstacle in achieving therapeutic efficacy. Till recently the TMZ resistance was mainly attributed to the repair activity of O6-methylguanine-DNA methyltransferase (MGMT) [22, 31, 32]. Interestingly, in our study, irrespective of the MGMT status, PRMT5 inhibition sensitized the GSCs to TMZ. Thus, suggesting that the PRMT5 inhibition-induced sensitization of GSCs to TMZ is MGMT-status-independent.

Owing to the extensive studies on TMZ-resistance in glioblastoma and other tumor types, researchers have identified non-MGMT related therapy resistance mechanisms such as the presence of intrinsically resistant glioma stem cell populations with an enhanced DNA repair ability [6, 24, 25], epigenetic alterations, dysregulated signaling cascades [26], thus adding additional complexity to TMZ resistance mechanism.

TMZ causes G2/M cell cycle arrest in tumor cells providing an opportunity for the cells to repair the damaged DNA and to blunt cytotoxic effect [27, 28]. Here we show that the treatment of PRMT5-inhibited GSCs disrupts the G2/M cell cycle checkpoint in the TMZ treated cells and denies the opportunity for TMZ treated cells to repair the damaged DNA (Fig. 3A-D). Emerging evidence underscores the critical role of HR in mediating resistance to TMZ in glioblastoma. The key HR genes are frequently overexpressed in glioblastoma cells [33]. Silencing of RAD51 has been shown to enhance glioblastoma sensitivity to TMZ [34], and augment the response to radiotherapy [35]. In addition to prior reports, our mechanistic study shows that PRMT5 inhibition blocks multiple DNA damage repair mechanisms in general and HR in particular (Figs. 2, 3,4). In this study, TMZ treatment induced the expression of the HR gene RAD51, underlining the involvement of HR in imparting TMZ resistance. With the inhibition of PRMT5, TMZ-induced RAD51 was subdued suggesting the role of PRMT5 in HR in the context of glioblastoma. This mechanistic finding linking PRMT5 to HR and HR to TMZ resistance provides a novel insight and potential solution to overcome TMZ resistance in glioblastoma because, in the combination treatment, as PRMT5 inhibition severely affects the HR repair pathway (Figs. 4 and 5), it sensitizes GSCs to TMZ treatment.

Further, our quest for in-depth mechanistic understanding resulted in finding the potential link between PRMT5, the FA pathway, and TMZ resistance. Previous studies have demonstrated that PRMT5 regulates FA pathway at the epigenetic level [30] or through the preservation of mRNA splicing fidelity [36]. Additionally, reports suggest the role of FA pathway and TMZ resistance [29]. This is the first study that confirms the involvement of the PRMT5-FA pathway axis in regulating TMZ-induced HR (Fig. 6). In the present study, we demonstrate that PRMT5 positively regulates FA pathway components, as evidenced by concordant changes at the mRNA and protein levels assessed by quantitative gene expression analysis and immunoblotting. However, the precise mechanism underlying this regulation remains unresolved. Specifically, it is unclear whether PRMT5 exerts its effects primarily through epigenetic control of transcription, regulation of pre-mRNA splicing, or a combination of both mechanisms. Further mechanistic studies will therefore be required to delineate the molecular basis by which PRMT5 regulates the FA pathway in the context of TMZ resistance in glioblastoma.

As PRMT5 is a druggable target for glioblastoma [14] many PRMT5 inhibitors have been developed. LLY-283 is a selective SAM-competitive nucleoside inhibitor of PRMT5, and it demonstrates good brain penetration and significantly prolongs survival in mice with orthotopic glioblastoma models [15]. The promising preclinical results suggest that LLY-283 could be a valuable therapeutic agent for treating glioblastoma and possibly other cancers.

Since our results show enhanced anti-tumor efficacy when LLY-283 is combined with TMZ, our study not only highlights the importance of LLY-283 for clinical use but also the potential solution to overcome TMZ resistance in cancerous conditions in general and glioblastoma in particular. Overall, this study is the first of its kind that delineates the mechanistic and clinical relevance of PRMT5 in TMZ resistance in glioblastoma.

## Materials and Methods

### Cell Culture

The patient-derived primary glioblastoma neurospheres (GSCs) GSC040815 and GSC082209 were developed as described previously [37], and GBM12 and GBM43 were obtained from Dr. Jann Sarkaria’s laboratory (Mayo Clinic, Rochester, MN) and were cultured as described in the supplementary data. Molecular characterization (Suppl. Figure 1) and subtype information are detailed in the supplementary data.

### LLY-283 and TMZ

LLY-283 (PRMT5 inhibitor) was purchased from Selleck Chemicals LLC (Houston, TX, Cat. # S8883), and TMZ was procured from Sigma-Aldrich (St. Louis, MO, Cat. # T2577-25MG) and were reconstituted as per the manufacturer’s recommendation.

### Cell-titer glo Assay

The CellTiter-Glo Luminescent Cell Viability Assay (Promega, Madison, WI, Cat. # G7571) was performed following the manufacturer’s protocol to assess cell viability and/or proliferation. Luminescence was measured using a Biotek FLx800 (Agilent Technologies) microplate reader.

### siRNA Transfection

GSCs were transfected with either control siRNA (Non-Target Scrambled, Ctrl, Cat.# D-001810-10-20) or PRMT5-target-specific siRNA (P5i) (Cat.# J-015817-08-0050) or FANCD2-target-specific siRNA (FANCD2i) (Cat.# L-016376-00-0005) (Dharmacon, Lafayette, CO, US) using RNAiMAX Lipofectamine (Cat.# 13778150) and Opti-MEM (Cat.# 13778150) (Invitrogen, Carlsbad, CA, USA) according to the manufacturer’s instructions.

### Cell cycle analysis

The Cell cycle analysis was performed as described in the supplementary data.

### Western Blot

Western blot analysis was conducted as described in the supplementary data.

### Caspase 3/7 Activity Assay

As described in the supplementary data, the assay was performed as per the manufacturer’s instructions.

### Neurosphere formation assay

GSCs were seeded in 96-well plates ranging from 15 to 1000 cells per well. Cells were treated with Ctrl (DMSO), LLY-283 (GSC040815 & GSC082209 with 50 µM and GBM12 & GBM43 with 3 µM), TMZ (GSC040815 & GSC082209 with 50 µM and GBM12 & GBM43 with 6 µM) or the combination of LLY-283 and TMZ. The formation of neurospheres was observed daily for 7 days. The number of neurospheres formed in each well was counted and plotted against the number of cells seeded.

### RNA-sequencing

GSCs were treated with LLY-283 (50 µM), TMZ (50 µM), or a combination of LLY-283 and TMZ. Twenty-four hours post-treatment, cells were collected, and RNA was extracted using the RNeasy Mini Kit (Qiagen, Cat. # 74104). RNA sequencing (RNA-seq) experiments were conducted as previously described [37], utilizing the NextSeq 1000/2000 P2 system. The biological significance of these genes was analyzed through Gene Ontology (GO) and Gene Set Enrichment Analysis (GSEA) [38, 39]. RNA-seq results have been deposited in the GEO database (https://www.ncbi.nlm.nih.gov/geo/query/acc.cgi?acc=GSE286560).

### qPCR-based HR Assay

The HR Assay Kit (Norgen Biotek, Ontario, Canada, Cat. # 35600) was used to assess HR efficiency following the manufacturer’s instructions and as described previously [37]. See the supplementary data for the details.

### γH2AX and RAD51 Foci Assay

The cells were treated with either a control (DMSO), LLY-283, TMZ, FANCD2i, or a combination of LLY-283 and TMZ or FANCD2i and TMZ and subjected to Foci assay as described in the supplementary data.

### Single Cell Alkaline Gel Electrophoresis (Comet Assay)

PRMT5-depleted or LLY-283-treated GSCs were treated with either vehicle (0.1% DMSO v/v), or TMZ, or the combination of (P5i + TMZ, LLY-283 + TMZ), and the comet assay was conducted as detailed in the supplementary data.

### qPCR

Please refer to the supplementary information.

### Schematic Diagrams

The Schematic diagrams (Fig. 2H, Fig. 6A, and Fig. 6F) were generated using the BioRender software program, Scientific Image and Illustration Software | BioRender (Toronto, Ontario, Canada).

### Intracranial injections

Ethics Statement: The animal study was conducted following UT Health San Antonio IACUC approval and guidelines. The details of the study have been described in the supplementary data.

### Immunohistochemistry (IHC)

IHC was conducted as described in the supplementary data.

### Statistical analysis

Experiments were performed in biological replicates; data shown represent technical replicates. The statistical analyses were performed using GraphPad Prism software as described previously [40] and detailed in the supplementary data. Error bars represent the standard deviation unless otherwise specified.

## Availability of data and material

RNA-seq results have been deposited in the GEO database (https://www.ncbi.nlm.nih.gov/geo/query/acc.cgi?acc=GSE286560)

## Supplementary information

Supplementary material

## References

[1] Stupp R, Mason WP, van den Bent MJ, Weller M, Fisher B, Taphoorn MJ, et al. Radiotherapy plus concomitant and adjuvant temozolomide for glioblastoma. N Engl J Med. 2005;352:987–96. 15758009 10.1056/NEJMoa043330

[2] Stupp R, Taillibert S, Kanner A, Read W, Steinberg D, Lhermitte B, et al. Effect of tumor-treating fields plus maintenance temozolomide vs maintenance temozolomide alone on survival in patients with glioblastoma: a randomized clinical trial. JAMA. 2017;318:2306–16. 29260225 10.1001/jama.2017.18718 PMC5820703

[3] Qazi MA, Vora P, Venugopal C, Sidhu SS, Moffat J, Swanton C, et al. Intratumoral heterogeneity: pathways to treatment resistance and relapse in human glioblastoma. Ann Oncol : J Eur Soc Med Oncol. 2017;28:1448–56. 10.1093/annonc/mdx169 28407030

[4] Szerlip NJ, Pedraza A, Chakravarty D, Azim M, McGuire J, Fang Y, et al. Intratumoral heterogeneity of receptor tyrosine kinases EGFR and PDGFRA amplification in glioblastoma defines subpopulations with distinct growth factor response. Proc Natl Acad Sci USA. 2012;109:3041–6. 22323597 10.1073/pnas.1114033109 PMC3286976

[5] Lathia JD, Mack SC, Mulkearns-Hubert EE, Valentim CL, Rich JN. Cancer stem cells in glioblastoma. Genes Dev. 2015;29:1203–17. 26109046 10.1101/gad.261982.115 PMC4495393

[6] Bao S, Wu Q, McLendon RE, Hao Y, Shi Q, Hjelmeland AB, et al. Glioma stem cells promote radioresistance by preferential activation of the DNA damage response. Nature. 2006;444:756–60. 17051156 10.1038/nature05236

[7] Balbous A, Cortes U, Guilloteau K, Rivet P, Pinel B, Duchesne M, et al. A radiosensitizing effect of RAD51 inhibition in glioblastoma stem-like cells. BMC Cancer. 2016;16:604. 27495836 10.1186/s12885-016-2647-9 PMC4974671

[8] Carruthers R, Ahmed SU, Strathdee K, Gomez-Roman N, Amoah-Buahin E, Watts C, et al. Abrogation of radioresistance in glioblastoma stem-like cells by inhibition of ATM kinase. Mol Oncol. 2015;9:192–203. 25205037 10.1016/j.molonc.2014.08.003 PMC5528679

[9] Ahmed SU, Carruthers R, Gilmour L, Yildirim S, Watts C, Chalmers AJ. Selective inhibition of parallel DNA damage response pathways optimizes radiosensitization of glioblastoma stem-like cells. Cancer Res. 2015;75:4416–28. 26282173 10.1158/0008-5472.CAN-14-3790

[10] Venere M, Hamerlik P, Wu Q, Rasmussen RD, Song LA, Vasanji A, et al. Therapeutic targeting of constitutive PARP activation compromises stem cell phenotype and survival of glioblastoma-initiating cells. Cell Death Differ. 2014;21:258–69. 24121277 10.1038/cdd.2013.136 PMC3890948

[11] Yan F, Alinari L, Lustberg ME, Martin LK, Cordero-Nieves HM, Banasavadi-Siddegowda Y, et al. Genetic validation of the protein arginine methyltransferase PRMT5 as a candidate therapeutic target in glioblastoma. Cancer Res. 2014;74:1752–65. 24453002 10.1158/0008-5472.CAN-13-0884 PMC3959215

[12] Han X, Li R, Zhang W, Yang X, Wheeler CG, Friedman GK, et al. Expression of PRMT5 correlates with malignant grade in gliomas and plays a pivotal role in tumor growth in vitro. J Neurooncol. 2014;118:61–72. 24664369 10.1007/s11060-014-1419-0 PMC4076054

[13] Banasavadi-Siddegowda YK, Russell L, Frair E, Karkhanis VA, Relation T, Yoo JY, et al. PRMT5-PTEN molecular pathway regulates senescence and self-renewal of primary glioblastoma neurosphere cells. Oncogene. 2017;36:263–74. 27292259 10.1038/onc.2016.199 PMC5240810

[14] Banasavadi-Siddegowda YK, Welker AM, An M, Yang X, Zhou W, Shi G, et al. PRMT5 as a druggable target for glioblastoma therapy. Neuro Oncol. 2018;20:753–63. 29106602 10.1093/neuonc/nox206 PMC5961180

[15] Sachamitr P, Ho JC, Ciamponi FE, Ba-Alawi W, Coutinho FJ, Guilhamon P, et al. PRMT5 inhibition disrupts splicing and stemness in glioblastoma. Nat Commun. 2021;12:979. 33579912 10.1038/s41467-021-21204-5 PMC7881162

[16] Briggs KJ, Cottrell KM, Tonini MR, Tsai A, Zhang M, Whittington DA, et al. TNG908 is a brain-penetrant, MTA-cooperative PRMT5 inhibitor developed for the treatment of MTAP-deleted cancers. Transl Oncol. 2025;52:102264. 39756156 10.1016/j.tranon.2024.102264 PMC11832951

[17] Clarke TL, Sanchez-Bailon MP, Chiang K, Reynolds JJ, Herrero-Ruiz J, Bandeiras TM, et al. PRMT5-dependent methylation of the TIP60 coactivator RUVBL1 is a key regulator of homologous recombination. Mol Cell. 2017;65:900–16 e7. 28238654 10.1016/j.molcel.2017.01.019 PMC5344794

[18] Wang YJ, Cao JB, Yang J, Liu T, Yu HL, He ZX, et al. PRMT5-mediated homologous recombination repair is essential to maintain genomic integrity of neural progenitor cells. Cell Mol Life Sci. 2024;81:123. 38459149 10.1007/s00018-024-05154-x PMC10923982

[19] Hamard PJ, Santiago GE, Liu F, Karl DL, Martinez C, Man N, et al. PRMT5 regulates DNA repair by controlling the alternative splicing of histone-modifying enzymes. Cell Rep. 2018;24:2643–57. 30184499 10.1016/j.celrep.2018.08.002 PMC6322662

[20] Owens JL, Beketova E, Liu S, Tinsley SL, Asberry AM, Deng X, et al. PRMT5 cooperates with pICln to function as a master epigenetic activator of DNA double-strand break repair genes. iScience. 2020;23:100750. 31884170 10.1016/j.isci.2019.100750 PMC6941881

[21] Hwang JW, Kim SN, Myung N, Song D, Han G, Bae GU, et al. PRMT5 promotes DNA repair through methylation of 53BP1 and is regulated by Src-mediated phosphorylation. Commun Biol. 2020;3:428. 32759981 10.1038/s42003-020-01157-z PMC7406651

[22] Ortiz R, Perazzoli G, Cabeza L, Jimenez-Luna C, Luque R, Prados J, et al. Temozolomide: an updated overview of resistance mechanisms, nanotechnology advances and clinical applications. Curr Neuropharmacol. 2021;19:513–37. 32589560 10.2174/1570159X18666200626204005 PMC8206461

[23] Jezierzanski M, Nafalska N, Stopyra M, Furgol T, Miciak M, Kabut J, et al. Temozolomide (TMZ) in the treatment of glioblastoma multiforme-a literature review and clinical outcomes. Curr Oncol. 2024;31:3994–4002. 39057168 10.3390/curroncol31070296 PMC11275351

[24] Chen J, Li Y, Yu TS, McKay RM, Burns DK, Kernie SG, et al. A restricted cell population propagates glioblastoma growth after chemotherapy. Nature. 2012;488:522–6. 22854781 10.1038/nature11287 PMC3427400

[25] Orzan F, De Bacco F, Crisafulli G, Pellegatta S, Mussolin B, Siravegna G, et al. Genetic evolution of glioblastoma stem-like cells from primary to recurrent tumor. Stem Cells. 2017;35:2218–28. 28895245 10.1002/stem.2703

[26] Li H, Chen L, Li JJ, Zhou Q, Huang A, Liu WW, et al. miR-519a enhances chemosensitivity and promotes autophagy in glioblastoma by targeting STAT3/Bcl2 signaling pathway. J Hematol Oncol. 2018;11:70. 29843746 10.1186/s13045-018-0618-0 PMC5975545

[27] Newlands ES, Stevens MF, Wedge SR, Wheelhouse RT, Brock C. Temozolomide: a review of its discovery, chemical properties, pre-clinical development and clinical trials. Cancer Treat Rev. 1997;23:35–61. 9189180 10.1016/s0305-7372(97)90019-0

[28] Hirose Y, Berger MS, Pieper RO. Abrogation of the Chk1-mediated G(2) checkpoint pathway potentiates temozolomide-induced toxicity in a p53-independent manner in human glioblastoma cells. Cancer Res. 2001;61:5843–9. 11479224

[29] Chen CC, Taniguchi T, D’Andrea A. The Fanconi anemia (FA) pathway confers glioma resistance to DNA alkylating agents. J Mol Med (Berl). 2007;85:497–509. 17221219 10.1007/s00109-006-0153-2

[30] Du C, Li SW, Singh SX, Roso K, Sun MA, Pirozzi CJ, et al. Epigenetic regulation of Fanconi anemia genes implicates PRMT5 blockage as a strategy for tumor chemosensitization. Mol Cancer Res. 2021;19:2046–56. 34521764 10.1158/1541-7786.MCR-21-0093 PMC9766387

[31] Rivera AL, Pelloski CE, Gilbert MR, Colman H, De La Cruz C, Sulman EP, et al. MGMT promoter methylation is predictive of response to radiotherapy and prognostic in the absence of adjuvant alkylating chemotherapy for glioblastoma. Neuro Oncol. 2010;12:116–21. 20150378 10.1093/neuonc/nop020 PMC2940581

[32] Hegi ME, Diserens AC, Gorlia T, Hamou MF, de Tribolet N, Weller M, et al. MGMT gene silencing and benefit from temozolomide in glioblastoma. N Engl J Med. 2005;352:997–1003. 15758010 10.1056/NEJMoa043331

[33] Short SC, Giampieri S, Worku M, Alcaide-German M, Sioftanos G, Bourne S, et al. Rad51 inhibition is an effective means of targeting DNA repair in glioma models and CD133+ tumor-derived cells. Neuro Oncol. 2011;13:487–99. 21363882 10.1093/neuonc/nor010 PMC3093331

[34] Quiros S, Roos WP, Kaina B. Rad51 and BRCA2-New molecular targets for sensitizing glioma cells to alkylating anticancer drugs. PLoS ONE. 2011;6:e27183. 22073281 10.1371/journal.pone.0027183 PMC3206939

[35] King HO, Brend T, Payne HL, Wright A, Ward TA, Patel K, et al. RAD51 is a selective DNA repair target to radiosensitize glioma stem cells. Stem Cell Rep. 2017;8:125–39. 10.1016/j.stemcr.2016.12.005 PMC5233453 28076755

[36] Gillespie MS, Chiang K, Regan-Mochrie GL, Choi SY, Ward CM, Sahay D, et al. PRMT5-regulated splicing of DNA repair genes drives chemoresistance in breast cancer stem cells. Oncogene. 2025;44:862–76. 39695328 10.1038/s41388-024-03264-1 PMC11932929

[37] Alejo S, Palacios BE, Venkata PP, He Y, Li W, Johnson JD, et al. Lysine-specific histone demethylase 1A (KDM1A/LSD1) inhibition attenuates DNA double-strand break repair and augments the efficacy of temozolomide in glioblastoma. Neuro Oncol. 2023;25:1249–61. 36652263 10.1093/neuonc/noad018 PMC10326496

[38] Subramanian A, Tamayo P, Mootha VK, Mukherjee S, Ebert BL, Gillette MA, et al. Gene set enrichment analysis: a knowledge-based approach for interpreting genome-wide expression profiles. Proc Natl Acad Sci USA. 2005;102:15545–50. 16199517 10.1073/pnas.0506580102 PMC1239896

[39] Mootha VK, Lindgren CM, Eriksson KF, Subramanian A, Sihag S, Lehar J, et al. PGC-1alpha-responsive genes involved in oxidative phosphorylation are coordinately downregulated in human diabetes. Nat Genet. 2003;34:267–73. 12808457 10.1038/ng1180

[40] Otani Y, Sur HP, Rachaiah G, Namagiri S, Chowdhury A, Lewis CT, et al. Inhibiting protein phosphatase 2A increases the antitumor effect of protein arginine methyltransferase 5 inhibition in models of glioblastoma. Neuro Oncol. 2021;23:1481–93. 33556161 10.1093/neuonc/noab014 PMC8408848

