# Supplementary material for: PRMT5 inhibition impairs Fanconi Anemia pathway-mediated homologous recombination and enhances the antitumor efficacy of Temozolomide in glioblastoma
Source: Cell Death Dis. 2026 Apr 15;17(1):505. doi: 10.1038/s41419-026-08739-5 (PMC13194734; doi:10.1038/s41419-026-08739-5)
Supplement: Supplementary file 1 — Supplementary material [file 41419_2026_8739_MOESM1_ESM.docx]

**PRMT5 inhibition impairs Fanconi Anemia pathway-mediated homologous recombination and enhances the antitumor efficacy of Temozolomide in glioblastoma**

Authors: Shumpei Onishi^1#^, Sridharan Jayamohan^2#^, Ashis Chowdhury^1#^, Sarah Rivas^1^, Yoshihiro Otani^3^, Celine Ertekin^1^_,_ Jean-Paul Bryant^1^, Sara A. Murphy^4^_,_ Kimberly A. Rivera-Caraballo^4^, Stuart Walbridge^1^, Bayu Sisay^5^, Dragan Maric^6^, Abdel Elkahloun^5^, Kory Johnson^7^, Desmond A. Brown^1^, John D. Heiss^1^, Ashish H. Shah^1^, Tae Jin Lee^3^, Sangamesh G. Kumbar^8^, Ji Young Yoo^3^, Andrew J. Brenner^9^, Balveen Kaur^4^, Gangadhara R. Sareddy^2,9^_,_ Yeshavanth Kumar Banasavadi-Siddegowda^1*^

*^1^*Surgical Neurology Branch, National Institute of Neurological Disorders and Stroke, National Institutes of Health, Bethesda, MD, USA *^2^*Department of Obstetrics and Gynecology, University of Texas Health San Antonio, San Antonio, TX, USA *^3^*Department of Neurosurgery, University of Texas Health Science Center at Houston, Houston, TX, USA *^4^*Georgia Cancer Center, Augusta University Medical Center, Augusta, GA, USA *^5^*Cancer Genetics Branch, National Human Genome Research Institute, National Institutes of Health, Bethesda, MD, USA. ^6^Flow and Imaging Cytometry Core Facility, NINDS, NIH, Bethesda, MD, USA
*^7^*Bioinformatics Core, Information Technology Program, NINDS, NIH, Bethesda, MD, USA *^8^*Department of Growth and Development, College of Dentistry, University of Nebraska Medical Center, Omaha, NE, USA. *^9^*Mays Cancer Center, University of Texas Health San Antonio, San Antonio, TX, USA.

# Contributed equally to this article

**Running Title:** PRMT5 inhibition enhances the anti-tumor efficacy of temozolomide * **Corresponding Author:** Yeshavanth Kumar Banasavadi-Siddegowda, 10 Center Drive, Room 3D03, Bethesda, MD 20892-1414. Phone: 301-451-0970; E-mail: [yesh.banasavadisiddegowda@nih.gov](mailto:yesh.banasavadisiddegowda@nih.gov) ***Competing interests:*** We have no competing interests to declare.

**Materials and Methods**

***Cell Culture*:** The patient-derived primary glioblastoma neurospheres (GSCs) GSC040815 and GSC082209 were developed as described previously (26), and GBM12 and GBM43 were obtained from Dr. Jann Sarkaria’s laboratory (Mayo Clinic, Rochester, MN). The cells were cultured as neurospheres in DMEM/F12 medium without phenol red (ThermoFisher Scientific, Waltham, MA, USA, Cat. # 21041025), supplemented with 1% penicillin-streptomycin (ThermoFisher Scientific, Waltham, MA, USA, Cat. # 15140122), 50 ng/mL fibroblast growth factor (FGF) (R&D System Inc., Cat. # 4114-TC-01M), 50 ng/mL epidermal growth factor (EGF), (R&D System Inc., Cat. # 236-EG-01M), 2% B-27 supplement without vitamin A (ThermoFisherScientific, Waltham, MA, USA, Cat.# 12587010), and 1% sodium pyruvate (ThermoFisher Scientific, Waltham, MA, USA, Cat.#11360070). Cultures were maintained in low-attachment flasks. Cells were dissociated using ACCUTASE (Innovative Cell Technologies Inc., San Diego, CA, USA, Cat. # AT-104) or TrypLE Express (ThermoFisher Scientific, Waltham, MA, USA, Cat. # 12604013), authenticated via short tandem repeat profiling, and screened for mycoplasma contamination, with all cultures testing negative. While GBM12, GSC040815, and GSC082209 are Mesenchymal, GBM43 belongs to the classical subtype.

***Western Blot*:** Cells were lysed with RIPA buffer (Sigma, St. Louis, MO, USA, Cat. # R0278) containing a protease/phosphatase inhibitor cocktail at 1X concentration (Cell Signaling, Danvers, MA, USA, Cat. # 5872S). Protein concentration was determined using the BioRad Protein Assay Kit (Bio-Rad, Hercules, CA, USA, Cat. # 5000112). Equal amounts of protein were denatured with 1X NuPAGE reducing agent and 1X NuPAGE LDS sample buffer, then loaded onto 4-12% Tris-Bis gels. Proteins were transferred to nitrocellulose membranes (ThermoFisher Scientific, Waltham, MA, USA, Cat. # IB23002). Antibodies against PRMT5 (Cat. # ab109451) and Tubulin (Cat. # ab6161) were obtained from Abcam (Cambridge, UK), while the antibody for H4R3 was procured from Epigentek (Farmingdale, NY, USA, Cat. # A-3718-100). Antibodies against PCNA (Cat. # 13110T), GAPDH (Cat. # 2118S), APEX1(Cat. #10519T), RAD23B (Cat. #13525T), RAD51(Cat. # 8875S), FANCD2 (Cat. #16323T) were purchased from Cell Signaling (Danvers, MA, USA) and POLD1(Cat. #ab186407) was purchased from Abcam Cambridge, UK). All antibodies were used at 1:1000 dilution.

***Immunohistochemistry:*** Xenograft tumor sections collected from the mice were incubated with Ki67 (Cell Signaling Inc, USA, Cat. # 12075S) (1:200), cleaved caspase-3 (Cell Signaling Inc, USA, Cat. # 9661S) (1:100), and γH2AX (Cell Signaling Inc, USA, Cat. # 2577S) (1:100) primary antibodies overnight, followed by secondary antibodies for 30 min. DAB substrate was used to detect the immunoreactivity. Tumor sections were then counterstained with hematoxylin. A Nikon Ti inverted microscope was used to capture the images. Ki67 and γH2AX were quantified as the number of positive cells in 5 randomly selected microscopic fields. The cleaved caspase-3 stain was quantified based on the intensity measured by IHC-profiler software using the NIH-ImageJ plugin.

***ɣH2AX and RAD51Foci Assay:*** GSCs were seeded onto Geltrex-coated (Thermo Fisher Scientific, Waltham, MA, Cat. # A1569601) Lab-Tek II chamber slides (Thermo Fisher Scientific, Waltham, MA) and incubated overnight. The cells were treated with either a control (DMSO), LLY-283, TMZ, or a combination of LLY-283 and TMZ. GSC040815 and GSC082209 were treated with 50 µM of TMZ and 50 µM of LLY-283. GBM43 and GBM12 were treated with 6 µM of TMZ and 3 µM of LLY-283. PRMT55-siRNA or FANCD2-siRNA-transfected cells were seeded onto Geltrex-coated chamber slides and treated with DMSO or TMZ. GSC040815 and GSC082209 were treated with 50 µM, and GBM43 and GBM12 were treated with 6 µM of TMZ. 48 hours post-treatment, the samples were fixed with 4% paraformaldehyde (Electron Microscopy Sciences, Hatfield, PA, Cat. # 15710-S) for 20 minutes and permeabilized with 0.1% Triton X-100 for 10 minutes. Immunofluorescence blocking buffer (Cell Signaling Technology, Danvers, MA, Cat. # 12727S) was used to block the cells for 1 hour at room temperature. The cells were then incubated overnight at 4 °C with γH2AX or RAD51 antibody (Cell Signaling Technology, Danvers, MA & Abcam, Cambridge, UK) at a 1:500 dilution. Slides were then incubated with Alexa Fluor 594-conjugated secondary antibody (Abcam, Waltham, MA, Cat. # ab150080) (1:200 dilution) for 1 hour at room temperature. Coverslips were mounted onto the slides using a vectashield antifade mounting solution with DAPI (Vector Laboratories, Newark, CA, Cat. # H-2000-10). Images of the γH2AX foci or RAD51 foci were captured using a confocal microscope (Leica Microsystems, Morrisville, NC), and the foci were counted manually.

***Single Cell Alkaline Gel Electrophoresis (Comet Assay):*** PRMT5-depleted or LLY-283-treated GSCs were treated with either vehicle (0.1% DMSO v/v), TMZ, or the combination of P5i + TMZ or LLY-283 + TMZ. For the LLY-283/TMZ treatment condition, GSC040815 and GSC082209 were treated with 50 µM of TMZ and 50 µM of LLY-283. GBM43 and GBM12 were treated with 6 µM of TMZ and 3 µM of LLY-283. GSC040815 and GSC082209 were treated with 50 µM, and GBM43 and GBM12 were treated with 6 µM of TMZ for PRMT5 knockdown experiment. Also, GSC040815 and GSC082209 were treated with 50 µM of TMZ. Forty-eight hours post-treatment, single-cell suspensions of GSCs were seeded in low-melting agarose (20×10^^5^ cells/mL) and 50 µl of the cell suspension was dispersed on the pre-treated microscope slides. Alkaline lysis (1 hour) and DNA unwinding (20 minutes) were performed before electrophoresis. Electrophoresis was done at 21V constant for 40 min in cold alkaline buffer. Samples were then dehydrated in 70% ethanol and air-dried for 15 minutes in the dark at 37°C incubator. Subsequently, the slides were stained with SYBR Gold (Comet assay kit, R & D Systems, Minneapolis, MN, Cat. # 4250-050-ESK) for 30 minutes. Comet images were captured using the EVOS fluorescence microscope. At least 20 representative comets were measured for each treatment group

***Cell cycle analysis*:** Cells were treated with respective treatment conditions. GBM43 and GBM12 were treated with 6 µM of TMZ and 3 µM of LLY-283. GSC040815 and GSC082209 were treated with 50 µM and GBM43 and GBM12 were treated with 6 µM of TMZ. After 48 hours, the cells were washed with phosphate-buffered saline (PBS) and fixed with 80% ethanol. Subsequently, the fixed cells were stained with 50 µg/mL propidium iodide (PI) (, Sigma-Aldrich, USA, Cat. # APOAF-60TST). Flow cytometric analysis was performed using a Becton Dickinson LSRII fluorescence-activated cell sorter (FACS) (Becton-Dickinson, San Jose, CA) and/or a MoFlo Astrios EQ cell sorter (Beckman Coulter, GA). Data were analyzed using Modfit software (Topsham, ME).

**Caspase 3/7 Activity Assay:** As described in the supplementary data, the assay was performed as per the manufacturer’s instructions. GSCs were seeded into 96-well plates and treated with LLY-283, TMZ, or the combination of LLY-283 + TMZ. GSC040815 and GSC082209 were treated with 50 µM of TMZ and 50 µM of LLY-283. GBM43 and GBM12 were treated with 6 µM of TMZ and 3 µM of LLY-283. 48 hours post-treatment, caspase 3/7 activity was assessed using the Caspase-Glo® 3/7 Assay System (Promega, Madison, WI, USA, Cat. # G8093) following the manufacturer’s instructions. For PRMT5-intact and depleted cells, 48 hours post-transfection, GSCs were seeded in 96-well plates and treated with increasing doses of TMZ. 48 hours post-treatment, caspase 3/7 activity was measured.

***qPCR-based HR Assay:*** The HR Assay Kit (Norgen Biotek, Ontario, Canada) was used to assess HR efficiency following the manufacturer’s instructions and as described previously (26). GSCs transfected with the HR kit plasmids using Lipofectamine 3000 (ThermoFisher Scientific, Waltham, MA, USA, Cat. # L3000008). Six hours post-transfection, the GSCs were treated with LLY-283 (50 µM), TMZ (50 µM), or the combination for 48 hours. Genomic DNA was then extracted using the QIAprep Spin Miniprep kit (Qiagen, Germantown, MD, USA, Cat. # 27104) according to the manufacturer’s protocol. Quantitative PCR (qPCR) was performed using the QuantStudio 6 Flex system (Life Technologies).

***RT-PCR:*** Post-treatment cells were pelleted, and RNA was purified (QIAgen RNeasy, Qiagen), per the manufacturer's instructions. Using SuperScript III First-Stand synthesis super mix (ThermoFisher Scientific, Waltham, MA, USA, Cat. # 11752050) cDNA was generated. mRNA expression was measured by quantitative RT-PCR (Mastercycler ep realplex, Eppendorf) using SYBR Green (Applied Biosystems). Gapdh was used as an internal control. Primer sequences: gapdh, forward, 5′-GGAGTCAACGGATTTGGTCG-3′; reverse, 5′-GGAATCATATTGGAACATGTAAACC-3′. FANCE, forward, 5’-TGTAGTCCCAGCCAGATGGA-3’; reverse, 5’-AAGAGGCTTCTGGTCAGCAC-3’. FANCD1, forward, 5’- ACTCTGCCGCTGTACCAATC-3’; reverse, 5’-GTGTCTGACGACCCTTCACA-3’. FANCD2, forward, 5’-CGACGGCTTCTCGGAAGTAA-3’; reverse, 5’-GGCATCTTCTGTCAGGCTC-3’. FANCA, forward, 5’-CTGGAAAGCCATCCCGACAT-3’; reverse, 5’-CCTGTACTCCAGCAGCCAAA-3’. FANCB, forward, 5’-CCGCTGCGTTGAGTTTCATA-3’; reverse, 5’- TCTGGGACAATAGGCATCACA-3’. FANCC, forward, 5’-AAGCCAATACGAGGCAAAGC-3’; reverse: 5’-GCACACATTAAATCGGGTGGT-3’.

***Intracranial injections*:** Ethics Statement: The animal study was conducted following UT Health San Antonio IACUC approval and guidelines. Animal studies: NOD.CB17*-Prkdc^scid^*/NCrCrl mice, aged 6-8 weeks were purchased from Charles River (Wilmington, MO). GFP-Luciferase expressing GSC040815 (GSC040815 GFP-Luc) (1X10^4^ cells/mice) were implanted in the mice intracranially. Day 4, post-tumor implantation, mice were randomized (by the lab member who was not part of the tumor implantation) to receive either vehicle (0.5% methylcellulose, 0.5% Tween 80 or 1:1 OraPlus: OraSweet), LLY-283 (50 mg/kg body weight/day in 0.5% methylcellulose, 0.5% Tween 80), TMZ (10 mg/kg body weight in 1:1 OraPlus: OraSweet ), or in combination via oral gavage. 50 mg/kg of LLY-283 was administered orally in weekly cycles of 3 days on, and 4 days off, until all the mice in the TMZ-treatment alone group reached the end stage of the study. Mice were treated with TMZ on days 7, 9, 11, 13, and 15 post-tumor implantation by oral gavage. The Xenogen IVIS system was used to follow the tumor growth. The mice were monitored regularly for neurological symptoms from the time of tumor implantation till they reached the experimental endpoint. Once they reached the end stage of the study, mice were euthanized and recorded their survival.

***Statistical analysis*:** Statistical analyses were performed using GraphPad Prism software. A two-sided unpaired Student’s t-test was employed to determine statistical significance between two continuous groups, with results presented as mean values ± standard deviation. Survival curves were plotted using the Kaplan-Meier method, and statistical significance was assessed with the log-rank test. The Benjamini-Hochberg procedure was applied to adjust for multiple comparisons in post-hoc analyses. To further assess the survival benefit of the LL-Y283 + TMZ combination, a Cox proportional hazards regression model was fitted, and hazard ratios were calculated as described previously (29). A p-value of <0.05 was considered indicative of statistical significance.

**Supplementary Figures**

**
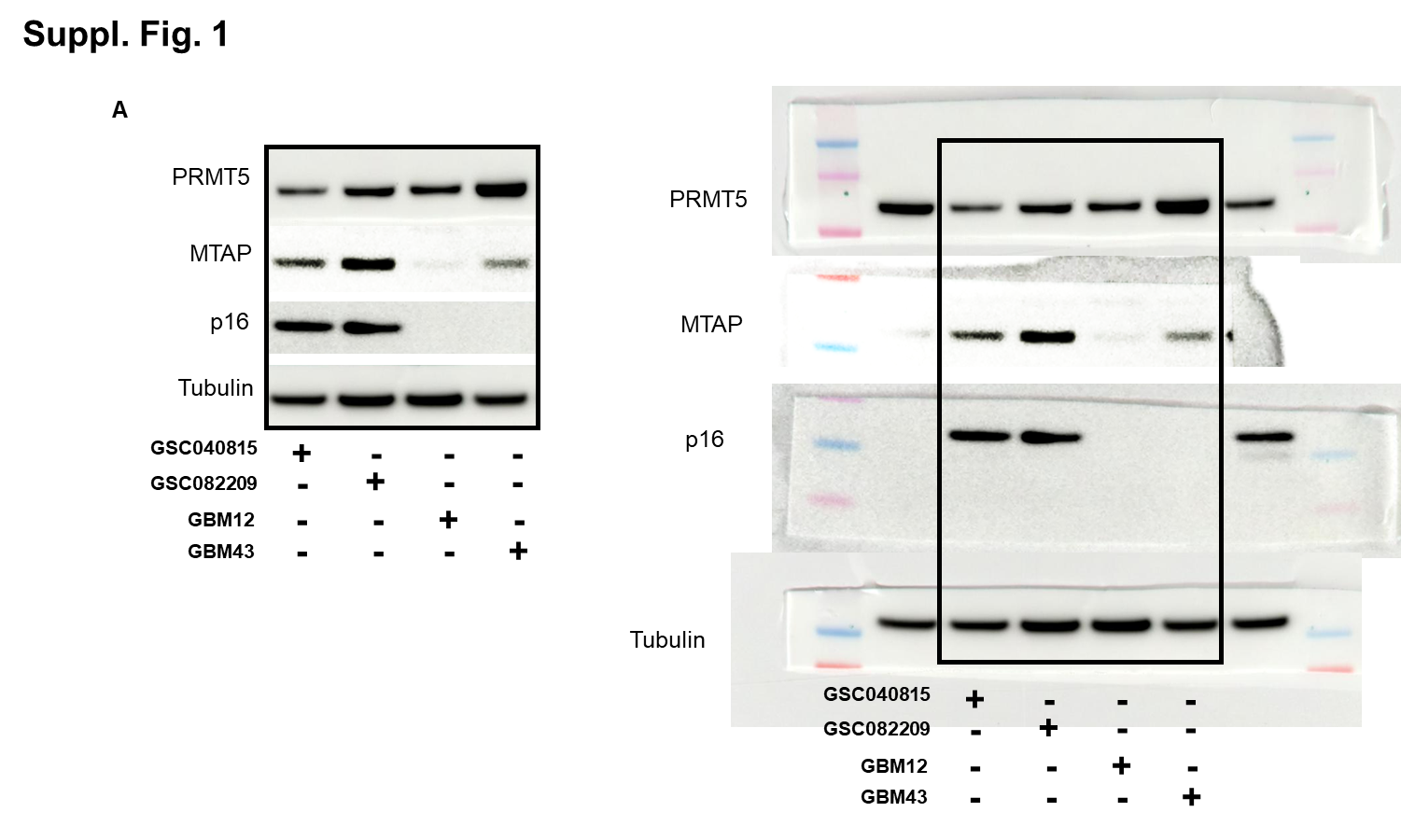
 Supplementary Figure 1:** Western blot images (cropped and uncropped) showing the PRMT5, MTAP & p16 status of the GSCs used for this study

.

**
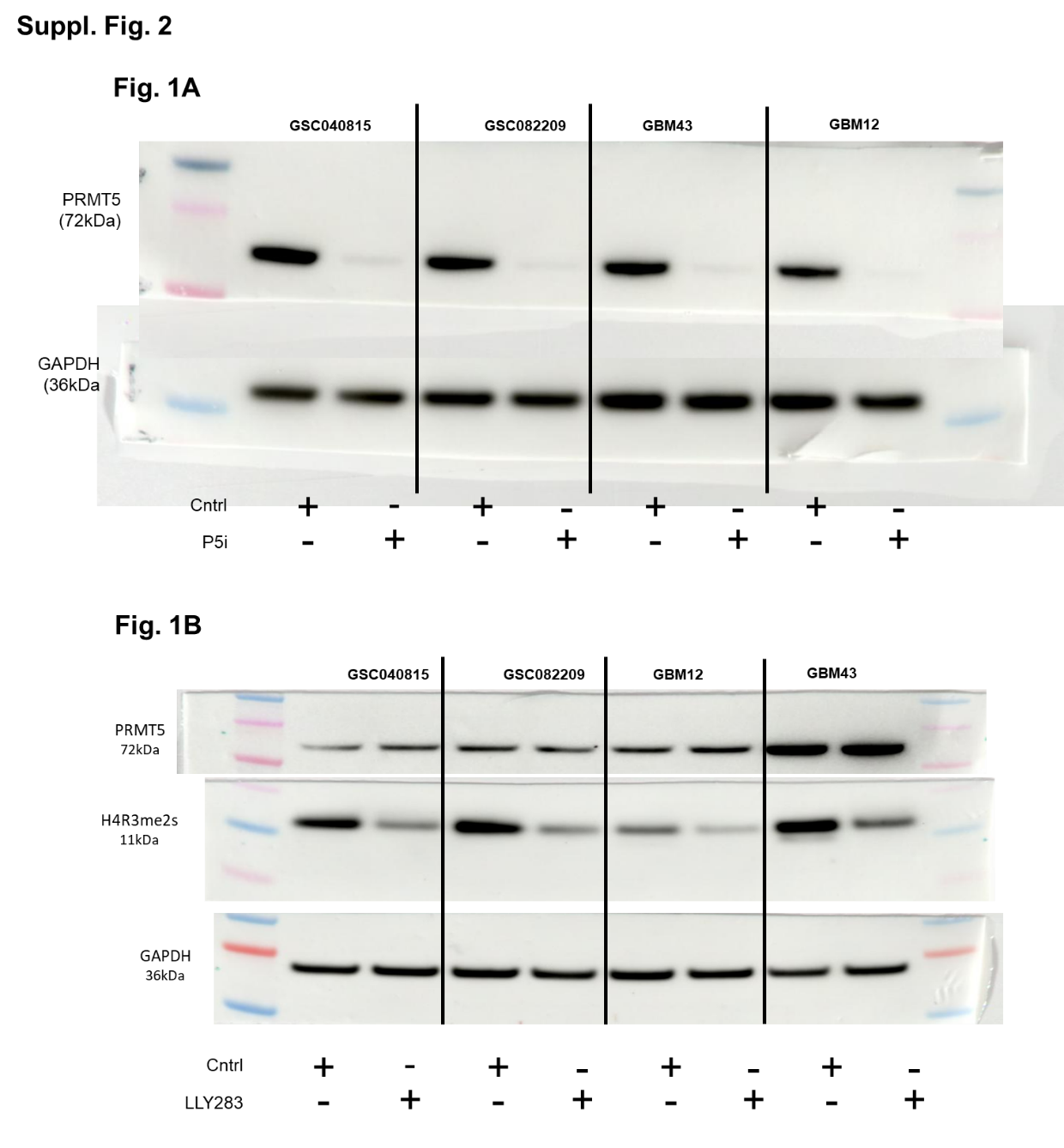
**

**Supplementary Figure 2:** Uncropped western blot image for Figure 1A and 1B.

**
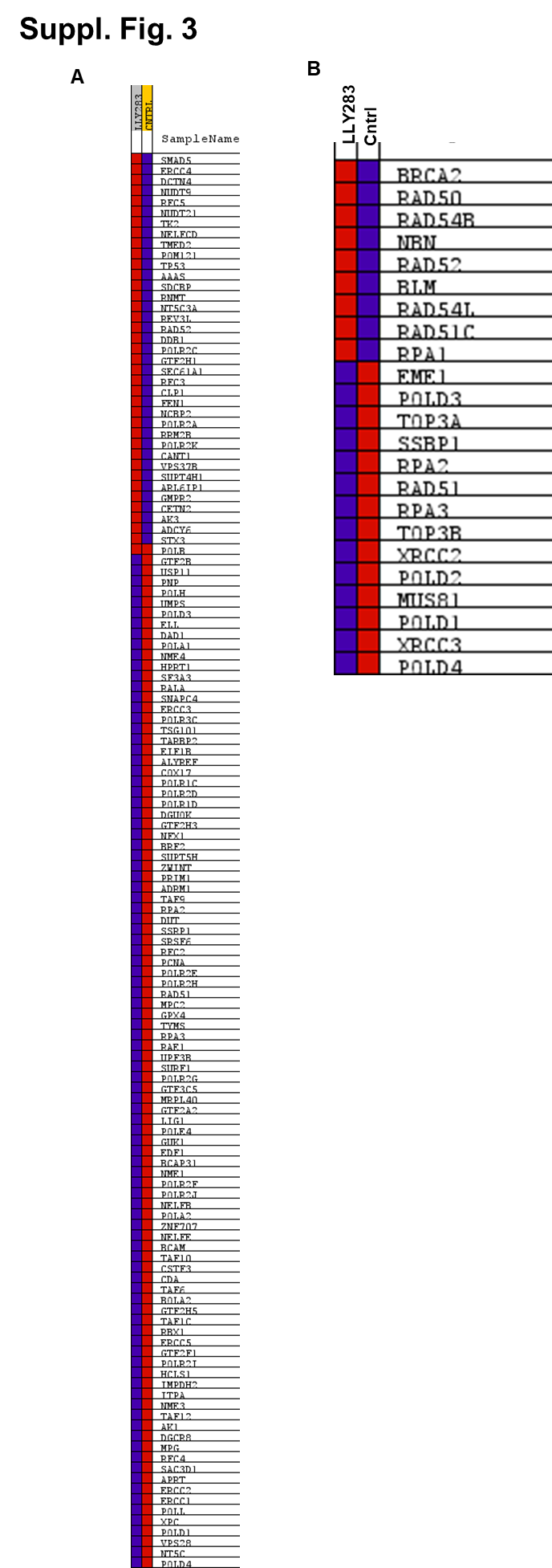
**

**Supplementary Figure 3: A.** Heatmap (related to Fig. 2A) showing the expression of DNA damage repair genes in Cntrl and LLY-283 treated GSC082209. **B.** Heatmap (related to Fig. 2E) showing the expression of HR repair genes in Cntrl and LLY-283 treated GSC082209.

**
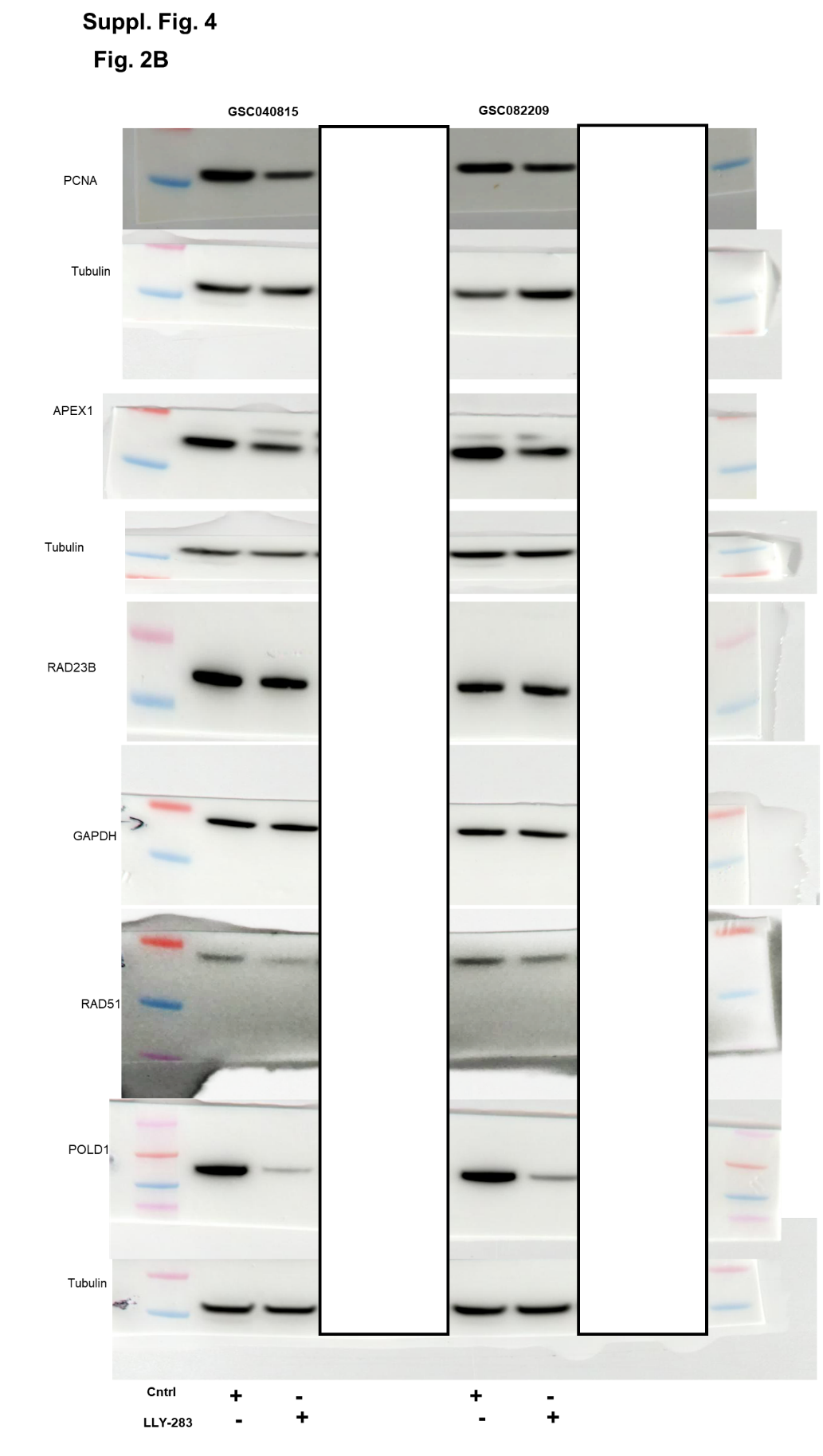
**

**Supplementary Figure 4: A.** Uncropped images of Western blot for Figure 2B.

**
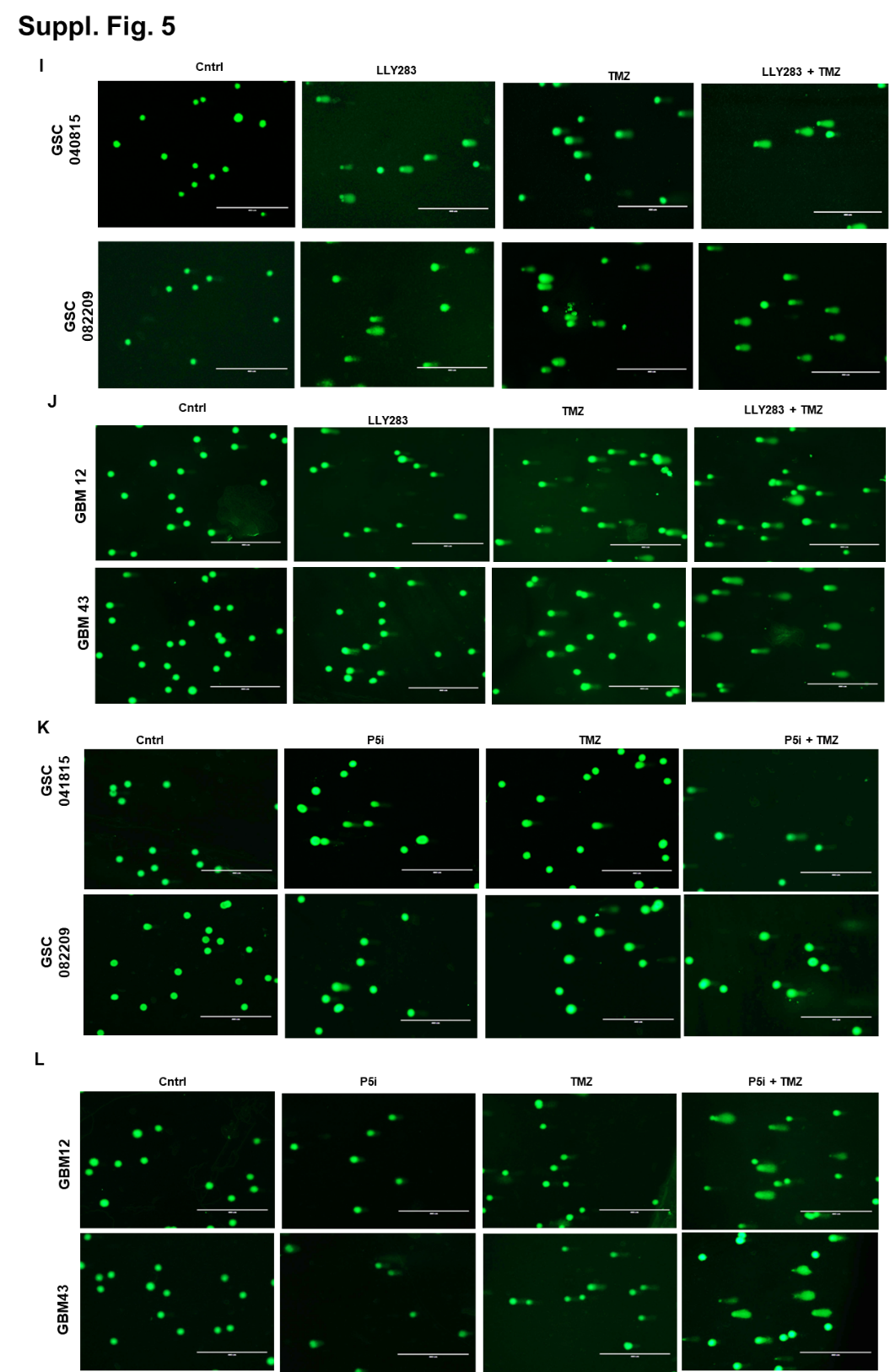
**

**Supplementary Figure 5: A.** Uncropped images of comet assay for Figure 3I-L

**
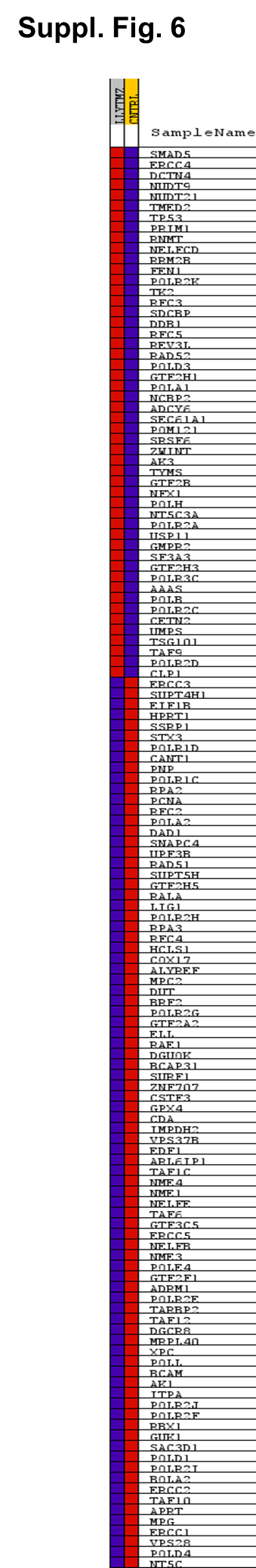
**

**Supplementary Figure 6:** Heatmap showing differential gene expression based on Fig. 4A.

**
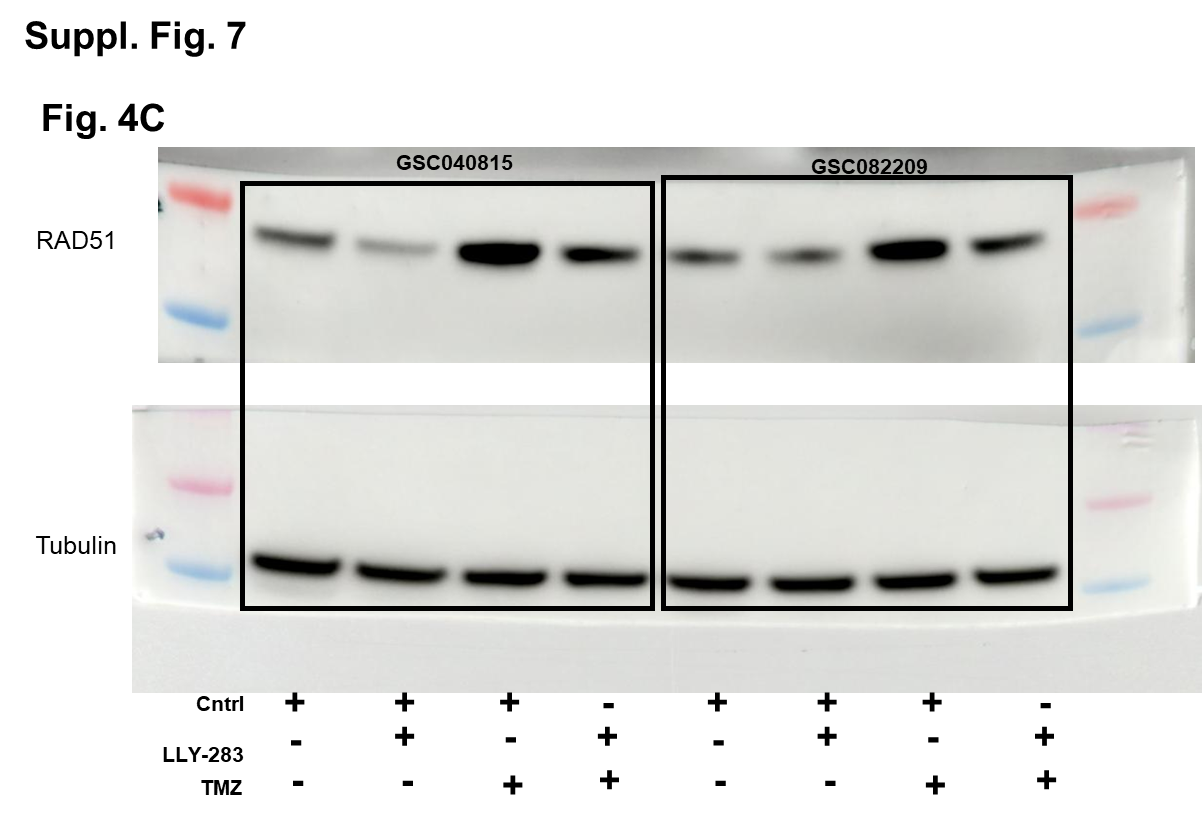
**

**Supplementary Figure 7: A.** Uncropped images of Western blot for Figure 4C.

**
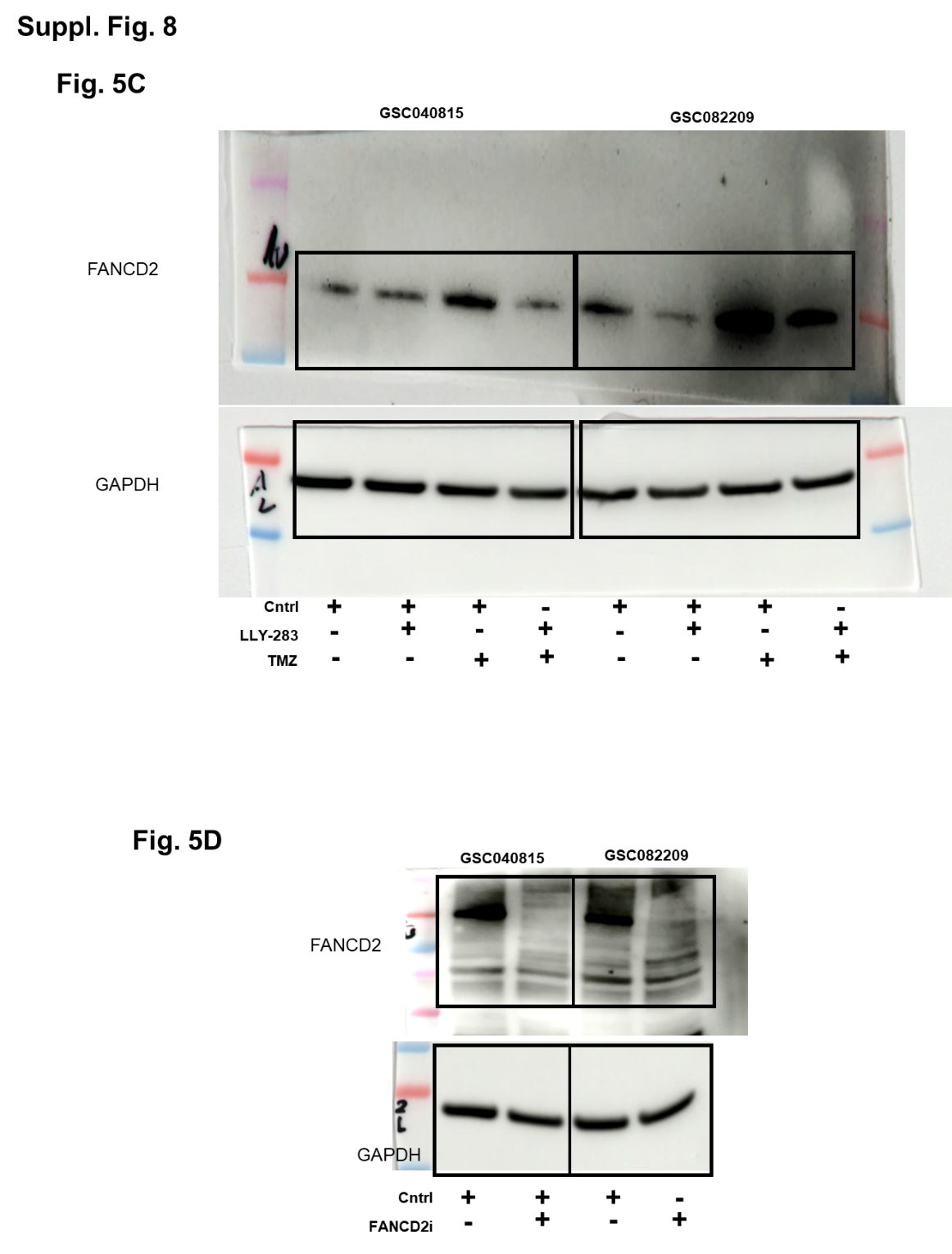
**

**Supplementary Figure 8: A.** Uncropped images of Western blot for Figure 5C & 5D.
